# The Importance of Non-Native Prey, the Zebra Mussel *Dreissena polymorpha*, for the Declining Greater Scaup *Aythya marila*: A Case Study at a Key European Staging and Wintering Site

**DOI:** 10.1371/journal.pone.0145496

**Published:** 2015-12-28

**Authors:** Dominik Marchowski, Grzegorz Neubauer, Łukasz Ławicki, Adam Woźniczka, Dariusz Wysocki, Sebastian Guentzel, Maciej Jarzemski

**Affiliations:** 1 Department of Vertebrate Zoology and Anthropology, Faculty of Biology, Szczecin University, Szczecin, Poland; 2 West–Pomeranian Nature Society, Szczecin, Poland; 3 Ornithological Station, Museum and Institute of Zoology, Polish Academy of Sciences, Gdańsk, Poland; 4 National Marine Fisheries Research Institute, Research Station in Świnoujście, Świnoujście, Poland; 5 Linus Maciej Jarzemski, Szczecin, Poland; CSIR- National institute of oceanography, INDIA

## Abstract

The European population of Greater Scaup *Aythya marila* has experienced an alarming, ~60% decline in numbers over the last two decades. The brackish lagoons of the Odra River Estuary (ORE) in the south-western Baltic Sea, represent an important area for the species during the non-breeding season in Europe. The lagoons regularly support over 20 000 Scaup, with peaks exceeding 100 000 (38%–70% of the population wintering in NW Europe and the highest number recorded in April 2011–105 700). In the ORE, Scaup feed almost exclusively on the non-native Zebra Mussel *Dreissena polymorpha*. This mussel was present in the ORE already in the 19^th^ century and continues to be superabundant. Using the results of 22 Scaup censuses (November to April 2002/2003 to 2013/2014) from the whole ORE (523 km^2^ of water), we show that Scaup flocks follow areas with the greatest area of occurrence and biomass of the Zebra Mussel, while areas with low mussel densities are ignored. The numbers of Scaup in the ORE are primarily related to the area of Zebra Mussel occurrence on the lagoon’s bottom (km^2^) in a non-linear fashion. Zebra Mussels were absolutely prevalent (97% of biomass) in the digestive tracts of birds unintentionally by-caught in fishing nets (n = 32). We estimate that Scaup alone consume an average of 5 400 tons of Zebra Mussels annually, which represents 5.6% of the total resources of the mussel in the ORE. Our results provide a clear picture of the strong dependence of the declining, migratory duck species on the non-native mussel, its primary food in the ORE. Our findings are particularly important as they can form the basis for the conservation action plan aimed at saving the north-western European populations of Scaup.

## Introduction

**Fig 1:**
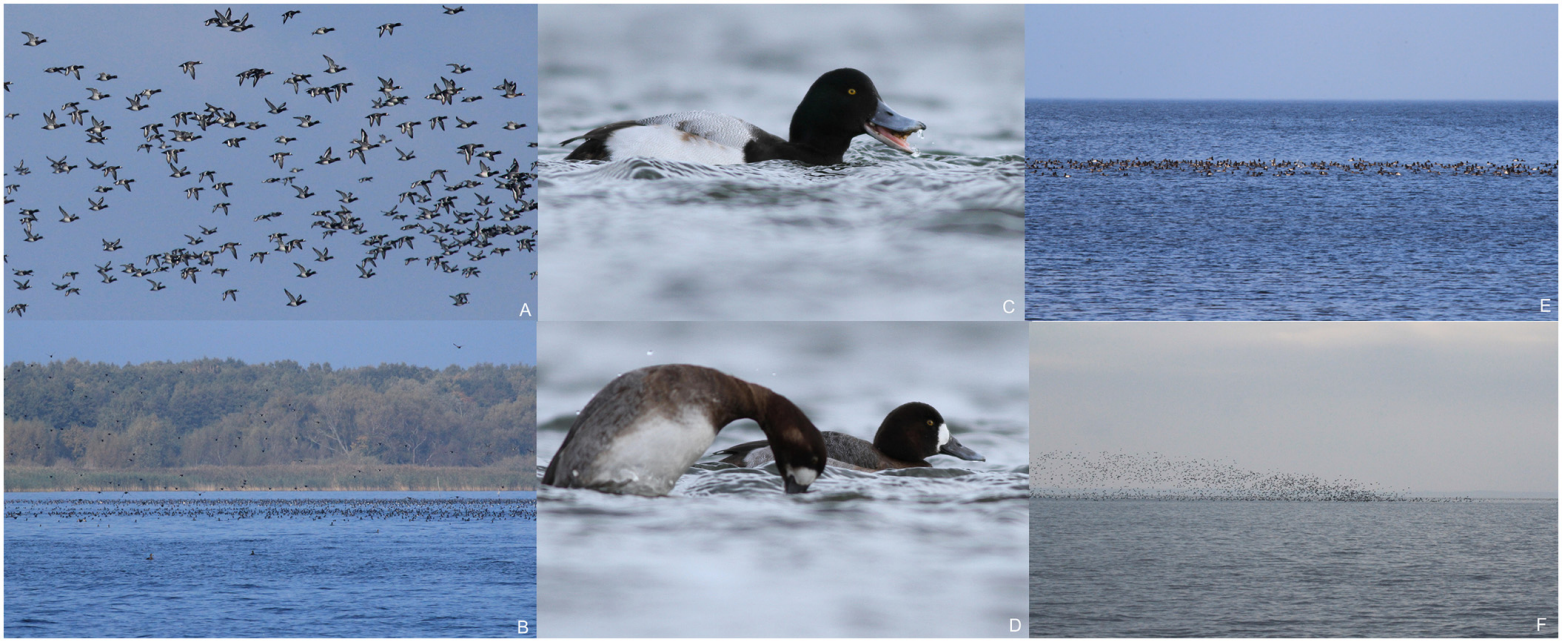
Greater Scaup *Aythya marila* and its flocks in the Odra River Estuary. A—Scaup flock in the Miroszewo (MI) subarea, 16.10.2013, photo by D. Marchowski. B and E—part of a line of Scaup directly above the Zebra Mussel aggregation in the Miroszewo (MI) subarea, B—16.10.2013, E—09.10.2013, photo by D. Marchowski. C—adult male of Scaup, Pomeranian Bay near Świnoujście, 16.02.2014, photo by D. Kilon. D—Scaup females in the Pomeranian Bay near Świnoujście, 16.02.2014, photo by D. Kilon. F—part of a 30 000-strong flock of Scaup directly above the Zebra Mussel aggregation in the Krzecki Wyskok (KW) subarea, 16.10.2013, photo by D. Marchowski.

The Eurasian subspecies of the Greater Scaup *Aythya marila marila* (hereafter Scaup) (Fig 1c and 1d) breeds across the boreal zone from Iceland east to the River Lena. About 95% of the population wintering in Europe originates from northern Russia (northern Europe and western Siberia). Key breeding grounds are situated in the lower Pechora River basin [1]. The main wintering grounds are in the lagoons and bays of the south-western Baltic Sea [2], and in the south-eastern North Sea [3]. Outside the breeding period, the Scaup occupies two ecological niches. It lives both on seawaters, like other ducks of the tribes *Somaterini* and *Mergini* [4, 5, 6], and on freshwater bodies like typical *Aythyini* [7, 8]. During migration and wintering, Scaup form mixed flocks with other sympatric species, frequently with Tufted Duck *A*. *fuligula* and Pochard *A*. *ferina* [9, 10, 11, 12, 13, 14]. The Odra River Estuary (hereafter ORE) is one of two areas, where the European population of Scaup congregates in the largest numbers during the non-breeding season [2, 3, 15]; the reasons for its high abundance here have remained unclear, however. Its main food are benthic bivalves (mainly mussels) [5] obtained by diving (Fig 1d). Like other ducks of the genus *Aythya*, Scaup forage mainly at night [6, 16, 17], but there are also studies documenting daytime feeding [8, 18]. In wintering areas, Scaup flocks regularly move between night-time foraging areas and daytime roosts; these movements were first described by Liepe from Germany [16, 17] and by Nilson [6, 19] from Scania (Sweden). Alternatively, birds may remain in the area without undertaking daily migrations [18]. The favored prey items in winter are marine or freshwater bivalves, whereas in spring these ducks regularly feed on fish eggs [4, 5, 8]. Previous studies outside the ORE have shown that Scaup wintering in the Baltic area forage mainly on marine bivalves, including Sand Gaper *Mya arenaria*, Blue Mussel *Mytilus edulis*, and Baltic Clam *Macoma balthica* [5, 6, 20]. On the Dutch lakes IJsselmeer and Markermeer their basic food is the freshwater Zebra Mussel *Dreissena polymorpha* [8], an alien species originating from the Black and Caspian Sea basins [21]. In view of the alarming, rapid decline of Scaup (up to 60% in the last 20 years [2, 15]), a comprehensive study of the species’ ecology is required in order to draw up appropriate conservation measures. In our work, we focused on the distribution and numbers of Scaup in the Polish part of the ORE, an area known for huge concentrations of the species [9, 10, 11, 12, 13, 14, 22, 23, 24, 25]. During waterfowl censuses we regularly observed large flocks of Scaup in some areas of the ORE, while in others there were few or no birds at all (Fig 1a, 1b, 1e and 1f). In some areas, we observed the characteristic shapes of Scaup flocks—long lines of ducks a few hundred meters from the shore (Fig 1b and 1e) or irregularly-shaped, dense flocks a few kilometers from the shore (Fig 1f). The ORE was colonized by Zebra Mussel in the mid-19^th^ century; nowadays it is still considered to be one of the sites with the largest aggregations of this freshwater mussel in central-western Europe [26]. We predicted that the abundance of Scaup flocks is positively related to the areas of Zebra Mussel aggregations on the water bodies bed. To test this prediction, we expressed the Zebra Mussel abundance by two measures related to abundance (area of occurrence and biomass) with extensive sampling in the ORE and then used bird census results to assess the strength of the relationship between Scaup abundance and Zebra Mussel area of occurrence and biomass. We also analyzed the digestive tract contents of dead birds caught in fishnets to confirm that the species does indeed feed on Zebra Mussel in the ORE.

More specifically, our aim was to test two alternative hypotheses: 1) Scaup use the ORE area to feed on freshwater Zebra Mussels, as in the Dutch lakes [8] or the Great Lakes of North America [7]; 2) the ORE lagoons are used by Scaup for purposes other than feeding, presumably as daytime roosts for birds feeding at sea during the night (~15 km to the north) on marine bivalves, as demonstrated earlier in three other areas in the Baltic where Scaup spend the winter [6, 16, 17, 19, 20]. If the first hypothesis holds, we expect higher numbers of Scaup in the areas with the largest concentrations of food resources, notably, the Zebra Mussel. If the second hypothesis is true, however, we should not expect any strong relationship between the numbers of Scaup and the area of occurrence and biomass of Zebra Mussels.

The question then arises as to why the birds would fly out to sea at night to feed, where the food lies at a greater depth and requires a several km long flight (the distance to the ORE), both of which are associated with increased costs. Confirmation of the first hypothesis, however, does not exclude the possibility that birds feeding on Zebra Mussels during the daytime will fly to feed at night on the open sea; a further analysis was therefore carried out.

The evidence for the use of ORE by Scaup as a foraging or roosting area is the food preference and the proportion of the different types of food consumed. The biomass proportion of freshwater and marine organisms found in the esophagus and stomach of Scaup could reflect the use of different feeding areas by birds. If in the esophagus and stomach of Scaup we find only freshwater organisms such as the Zebra Mussel, or the proportion of freshwater organisms is high, this would support the idea that the birds feed mainly in the ORE. Conversely, a high biomass of Baltic marine organisms (such as Sand Gaper, Blue Mussel or Baltic Clam) found in the digestive tracts would suggest that Scaup feed at sea and the ORE is used as a roosting site.

The ORE has a complicated structure of banks, and there are larger and smaller coves, some sheltered from the wind, others exposed to it. Therefore, if we assume that the Scaup use the ORE as a roosting site and forage elsewhere, we would expect them to choose sheltered areas with little wave action. In the following sections of our work, we have divided the subareas into sheltered and exposed ones in order to compare the numbers of Scaup recorded there. The above reasoning is expected to be correct if shelter is the prime factor driving the diurnal distribution of Scaup (the impacts of other factors, like predation pressure and disturbance from water sports, are probably distributed in equal measure between sheltered and exposed areas).

## Material and Methods

### Study area

**Fig 2:**
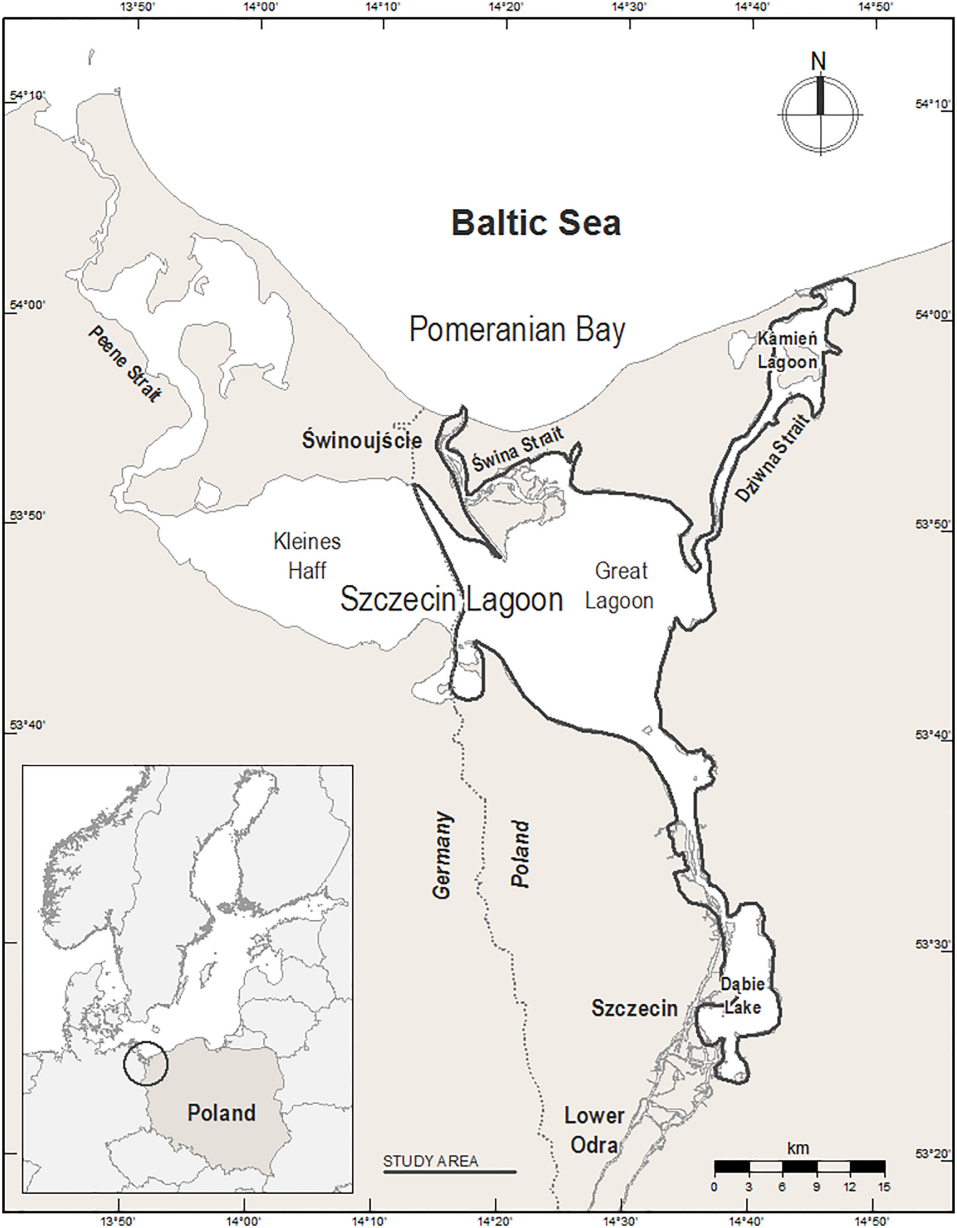
The study area—the Odra River Estuary, NW Poland, bordered by a bold line.

**Fig 3:**
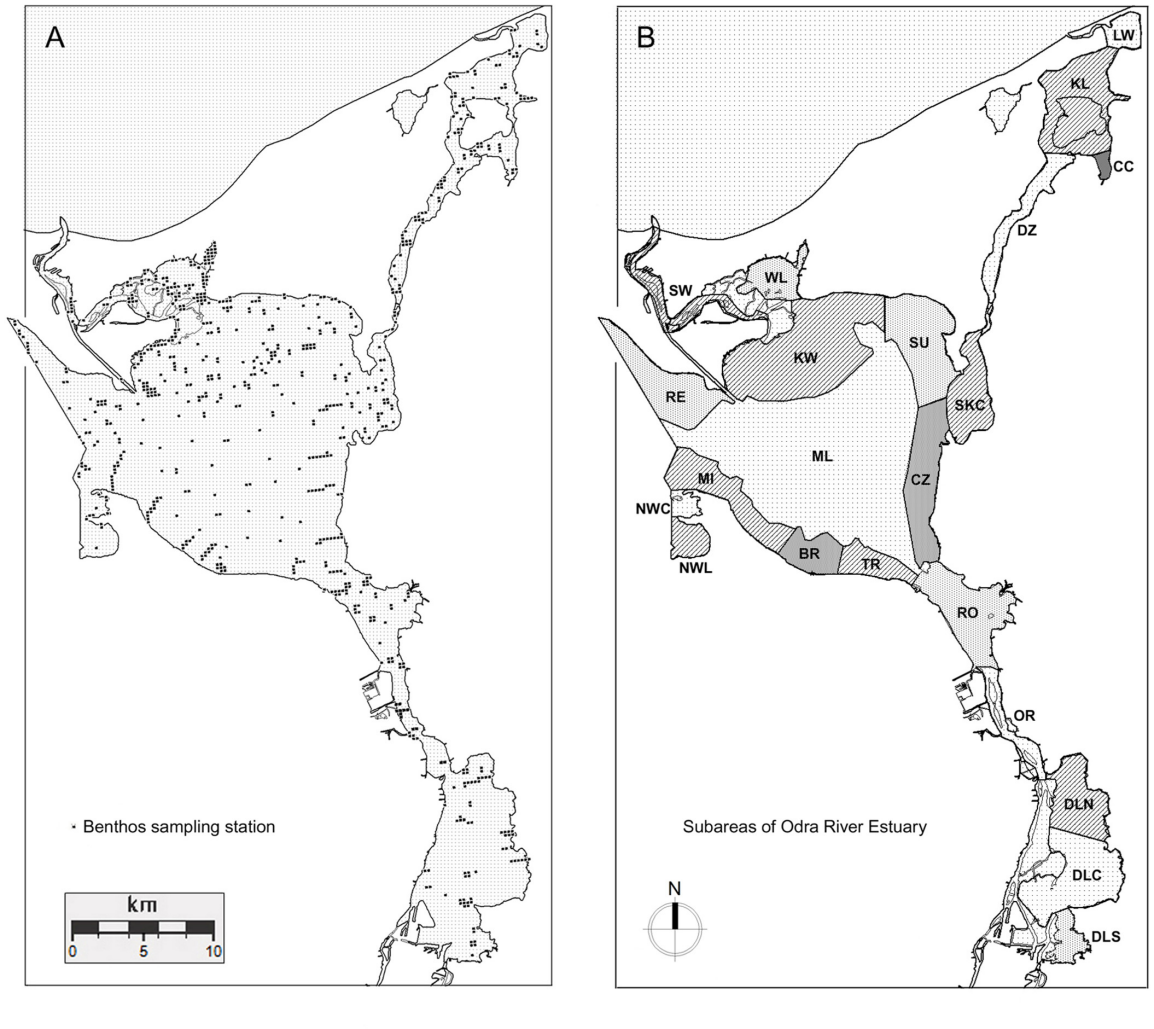
Location of benthos sampling stations (A) and the 22 Scaup census subareas (B).

The study area is located in the south-western part of the Baltic Sea and forms the Polish part of the ORE system, which includes the Great Lagoon (the Polish part of the Szczecin Lagoon), Świna Backward Delta, Kamień Lagoon, Dziwna Strait and Lake Dąbie; it covers a total area of 522.58 km^2^ (Fig 2, Table 1) The average and maximum depths of the Lagoon are 3.8 and 8.5 m, respectively (the dredged shipping lane cutting across the Lagoon from Baltic Sea to the port of Szczecin is 10.5 m deep) [27]. The waters of the Szczecin Lagoon, Kamień Lagoon and Lake Dąbie are brackish. The salinity in the central part of the Lagoon varies from 0.3 psu to 4.5 psu (mean = 1.4 psu) and declines with increasing distance from the sea [27]. Periodic inflows of water from the Pomeranian Bay (salinity ~7 psu) take place through the Świna Strait and, to a lesser extent, through the Dziwna and Peene Straits (the latter in the German part of the ORE). The Odra estuary is subject to strong anthropogenic pressure, which is manifested by a high level of eutrophication with all its adverse effects [27]. Communities of benthic organisms are typical of freshwater bodies and the fauna is characterized by a large proportion of Zebra Mussels, which have occurred here since the mid-19^th^ century. By the 1960s, the biomass of the Zebra Mussel in the Szczecin (Great) Lagoon was estimated at 110 000 tons [28, 29, 30]. In accordance with its geomorphological features, we divided the whole censused ORE area into 22 subareas, where birds were counted separately (Table 1, Fig 3b). In order to compare the mean number of birds in subareas differing in their exposure to wind and wave action, we further subdivided them into two groups: sheltered from and exposed to wind and wave action. During the study we noted which areas were exposed to strong wave action and which remained calm in the same weather. The sheltered areas are usually comparatively small coves surrounded by land; the exposed ones generally have a straight shoreline and are open to large water basins (see Table 1 and Fig 3b).

**Table 1:** Subareas of the Odra River Estuary, where Scaup *Aythya marila* were counted separately during the study period (2002–2014). (1) Number of subarea; (2) Name of subarea (the abbreviations used on the maps and charts are given in parentheses); (3) surface area of subarea in km^2^; (4) S—subarea sheltered from wind and wave action, E—subarea exposed to wind and wave action.

No (1)	Name of subarea (2)	area (km^2^) (3)	Sheltered (S) / exposed (E)(4)	Geographic coordinates of the subarea central point
1	Lake Wicko (WL)	11.06	S	N53.88; E14.40
2	Świna River (SW)	12.75	S	N53.86; E14.30
3	Skoszewska Cove (SKC)	16.70	E	N53.78; E14.59
4	Sułomino (SU)	28.40	E	N53.82; E14.55
5	Krzecki Wyskok (KW)	57.93	E	N53.82; E14.42
6	Main Lagoon (ML)	154.70	E	N53.76; E14.43
7	Refulat (RE)	23.45	E	N53.81; E14.28
8	Nowowarpieńska Cove (NWC)	3.32	S	N53.73; E14.29
9	Lake Nowowarpieńskie (NWL)	6.42	S	N53.71; E14.29
10	Miroszewo (MI)	20.40	E	N53.74; E14.33
11	Brzózki (BR)	11.95	E	N53.69; E14.41
12	Trzebież (TR)	11.05	E	N53.68; E14.47
13	Czarnocin (CZ)	24.50	E	N53.74; E14.53
14	Roztoka (RO)	29.10	E	N53.64; E14.57
15	Odra River (OR)	9.20	S	N53.56; E14.60
16	Lake Dąbie—North (DLN)	19.10	E	N53.50; E14.67
17	Lake Dąbie—Center (DLC)	29.50	E	N53.45; E14.67
18	Lake Dąbie—South (DLS)	7.80	S	N54.41; E14.65
19	Dziwna (DZ)	12.60	S	N53.91; E14.66
20	Kamień Lagoon (KL)	25.30	E	N53.98; E14.73
21	Lake Wrzosowo (LW)	6.05	E	N54.02; E14.79
22	Cicha Cove (CC)	1.30	S	N53.93; E14.75
	**Total**	**522.58**		

Most of the study area is part of the Natura 2000 network of European protected areas [31]. The authorities responsible for this area are the Maritime Office in Szczecin, Poland (Szczecin Lagoon and Kamień Lagoon), and the Regional Environmental Conservation Directorate in Szczecin, Poland (Lake Dąbie). As the whole area where we conducted the survey is freely accessible to the public, there was no need for a special permit.

### Spatial distribution and biomass of Zebra Mussels

The spatial distribution and biomass of Zebra Mussels were determined on the basis of extensive sampling during 2003–2004 and 2013–2014. We collected samples from the ORE bottom using standard samplers (0.1 m^2^ Van Veen and 0.225 m^2^ Ekman grabs, a 24 cm^2^ hand operated corer). At each sampling point we collected 1–10 subsamples which, when pooled, formed an integrated sample. In 2003–2004 the samples were sieved on a 0.8 mm mesh sieve, and all the mussels were counted and measured. The raw weight (with shells) of Zebra Mussels was determined for each sample using the Wolnomiejski and Witek [32] equation: W = 0.1487 × L^2.946^, where L is the shell length (mm) and W the weight of the individual mussel (mg). The raw weight includes the water contained in the mantle cavity, which is not a part of any tissue and accounts for about 20% of the weight [32]. Thus, subtraction of this mass from W yields the wet weight of a bivalve with shell [32]. In 2013–2014 our studies focused on identifying the Zebra Mussel’s distribution in the area. The Zebra Mussel samples from this later period were sieved on a 0.8 mm mesh sieve and weighed on an electronic balance. The mean mass of water from inside the shell, i.e., 20% was then subtracted from this value [32]. In 2003–2014 we obtained a total of 678 integrated samples (Fig 3a, S1 Table). Most of the quantitative data on biomass were collected in 2003–2004 (457 samples), so Zebra Mussel biomass data relate to that period. In 2013–2014 221 further samples were collected to confirm the fact that the Zebra Mussel in the ORE occurs in particular, more or less fixed, areas. The whole ORE was overlain with a grid of 2 × 2 km squares in MapInfo 8 software, which yielded 209 sample plots of 4 km^2^ (S1 Fig). The squares including the shoreline and the Polish–German border were smaller (water area of 0.06–3.9 km^2^). For each square, on the basis of sampling points (up to 20 sampling stations per square) we determined the occurrence range of mussels and their biomass averaged across sampling points within a square. Then, based on the mean biomass and the area occupied we calculated the biomass of mussels in each square. Samples were collected in the majority of squares (86%); only a small proportion were not sampled (29 squares, 14%). In the absence of samples from a given square, the biomass was determined by extrapolation, using the mean values of the adjacent squares, and the range was continued from adjacent squares. We then summed the biomass and area of occurrence of Zebra Mussels in each of the 22 Scaup counting subareas for the purpose of the analysis at the subarea level (see S2 Table, S1 Fig).

No specific permits were required for collecting the Zebra Mussels used for this study, because in Poland this species is neither privately owned nor protected in any way. The geographical coordinates of the benthos sampling station locations where we collected Zebra Mussels for each sample are given in S1 Table.

### Scaup censuses

Censuses were conducted using the standard methods for waterbird counts during the non-breeding season in large water bodies [33, 34]. Birds were counted during nine seasons (2002/2003 to 2013/2014), during the migration and wintering periods between November and April (see below). Three types of count methods were applied: from the shoreline, from aircraft and from boats. We compared different survey platforms in the ORE (aircraft, land and boat) and showed that the differences in counts for species staying on open water and forming flocks like Scaup are relatively small and that the figures are comparable (authors unpublished data DM, ŁŁ, SG). The detailed methodology and the results of all the counts are given elsewhere [9, 10, 12–14]. During the study period (2002/03–2013/14) we conducted 36 Scaup censuses for the whole ORE, while a further 13 were incomplete to a varying degree (2–18 subareas counted instead of 22, see S3 Table). For modeling the relationship between Zebra Mussel and Scaup, we selected only complete counts (all subareas counted) performed within 5 days or less, during good weather conditions; we also omitted the counts done during cold periods with 80% or more of the water frozen over. We avoided counting during bad weather (fog, heavy rain, strong wave action, strong wind) so as to reduce the possibility of overlooking large parts of the bird flocks. Otherwise, bad weather conditions such as strong wind or wave action would be an additional factor in determining the presence of birds in the area instead of food, on which this work was focused. This reduced the number of censuses to be analyzed to 22: ten were from an aircraft, two from a boat and the other ten from the shoreline. Nonetheless, some results from incomplete censuses were used for other analyses performed at the subarea level, e.g., comparison of bird numbers in exposed and sheltered areas (S3 and S4 Tables). The number of counts in each subarea ranged from 26 to 37 (S4 Table).

No specific permits were required for censuses performed in the study area in Poland. All the sections and observation points from which we counted the birds are located in freely accessible public areas.

### Modeling the relationship between Scaup density and Zebra Mussel abundance

Nine of the 22 counts selected for analysis took place in autumn (November), four in winter (January) and nine in spring (including seven in March and two in April). In order to investigate the relationship between Scaup numbers and Zebra Mussel abundance, we tested a range of models that account for correlated responses in R [35]. In our case, repeated counts probably leading to permanently similar (correlated) results were performed within subareas (between and within consecutive winters), within consecutive winters (counts during autumn, winter and spring within the same winter season) and at times within seasons (i.e., Scaup were counted twice per spring within a given winter season). Because initial data exploration suggested the presence of non-linear patterns between the response (Scaup densities in each of the 22 ORE subareas during consecutive censuses) and predictors, we used generalized additive mixed models with negative binomial error variance (NB GAMMs) in the library mgcv [36, 37, 38]. Initially, we tested the Poisson error variance, suited to count data, but it produced a large (>100) overdispersion. We therefore switched to negative binomial variance, as recommended by Zuur et al. [37] and Zuur [38]. We tested various biologically relevant combinations of random effects to find the most appropriate one; selection of the best-supported structure was based on AIC values. After the optimal structure was found, we followed the usual procedure of simplifying the model by the stepwise removal of insignificant effects. In order to reach the minimal adequate model according to the principle of parsimony, we applied *a posteriori* stepwise deletion of non-significant levels of the winter factor by aggregating them [37, 38]. We continued model selection until only significant terms were left in the model and/or the removal of any of them led to an increase of AIC.

The beyond-optimal NB GAMM included three factors included as either fixed or random effects: season (SEA) identifying the phenological period (autumn, winter, spring) within a given winter, winter (WIN), a factor identifying each of the eight winter seasons, and subarea (SAR), a factor identifying ORE subareas. Three continuous predictors, uncorrelated to each other, were the surface area of a given subarea (Area, expressed in km2), surface area of Zebra Mussel occurrence (Dpa, in km2) and density of the Zebra Mussel (Dpd, tons/1 km2, derived as the total Zebra Mussel biomass in a subarea divided by its area) in a given subarea, all included as smoothing functions. We didn’t use subarea depth since it shows a nonlinear association with both measures of the mussel abundance (area of occurrence and biomass, both of which were included in the model). Both Scaup and Zebra Mussel follow similar depths (2–4 m in case of mussel and ca 1.7–4 m in case of Scaup) (S2 Fig).

### Food composition

Dead birds drowned in gillnets (by-catch) were obtained from fishermen in ORE ports, from nets set on the Szczecin Lagoon and Lake Dąbie between November and April. Between 2008 and 2013 samples from 38 Scaup were collected and analyzed. Six of the dead birds had an empty stomach and esophagus, so they were discarded. The remaining 32 were dissected and thoroughly examined in the laboratory. All the items present in the digestive tracts (esophagus, gizzard and proventriculus) were identified to the lowest possible taxonomic level, using a stereomicroscope where necessary. All identifiable food items were counted and weighed. The food sample frequency of occurrence (%FO) was determined as the percentage of samples containing a given prey type. The dominance of biomass (%DB) was determined as the percentage biomass of a given prey type in the sample. The fraction of birds caught with mussels in the esophagus was used as the index of feeding success [39]. To determine the pattern of Scaup behavior in the ORE during foraging, i.e., whether the birds search specifically for Zebra Mussel or for different types of food, we compared the biomass of Zebra Mussels to the biomass of the other organisms found in the digestive tracts, at the level of individual birds. Within the Zebra Mussel aggregations in the ORE there are other organisms, such as *Chironomidae* larvae, leeches and snails [32], so even if Scaup consume primarily Zebra Mussels in the ORE, one can expect to find a fraction of other prey. In this case, the biomass of this “other taxa” fraction ought to be correlated with the Zebra Mussel biomass consumed, i.e., it should rise proportionally with increasing Zebra Mussel biomass. A positive and significant correlation would therefore indicate the Scaup generally do not specifically search for food other than Zebra Mussels.

### Calculation of yearly food consumption

The biomass of benthic animals consumed yearly by Scaup in the ORE was estimated on the basis of the numbers of these birds and the duration of their stay in the area. The number of bird-days was calculated as the seasonal mean number of birds multiplied by the average staging time (180 days). Calculations of daily energy demand and assimilation rate of the main prey were based on Stempniewicz & Meissner [20] and De Leeuw [8], and the local food composition was based on the food composition analysis reported in the previous paragraph. According to the calculations of Stempniewicz & Meissner [20], which take into account the energy content of the mussels (1.2 kJ/g bivalve wet mass), the assimilation ability of birds (0.84 kJ from 1 g of wet mass with shell) and the daily energy demand of 1 500 kJ for small ducks, the daily consumption of mussels should be 1 786 g FM (fresh mass) with shells but without the water from inside the mussel [20]. After experiments with trained birds in the Netherlands, De Leeuw [8] calculated that the mean daily requirement of Scaup is the equivalent of 2 240 g FM with shells. The differences result from the different method of measuring the wet mass. The Stempniewicz & Meissner [20] result applies to fresh mass without the water in the mantle cavity of mussels; De Leeuw [8] did not mention this, however, so we can assume that the fresh mass in this case was calculated with water. To obtain the real fresh mass of mussel with shell we need to subtract c. 20% of the water present in the mantle of the shell [32]. So if we subtract 20% from the 2 240 g FM calculated by De Leeuw, we get 1 792 g FM, a value nearly identical to the one calculated by Stempniewicz & Meissner. Therefore, in our further calculations we assumed the value of 1 790 grams of fresh mass (without water) to represent the daily food intake of Scaup.

### Ethics statement

This study complies with Polish regulations regarding the ethical treatment of research objects. No permits or approvals were needed for observational and fieldwork in the study area, because all of it is freely accessible public land. No protected species were sampled specifically for this study. The study was not carried out on private land. The only aspect of this study requiring a permit was for the acquisition of already dead (by-caught in fishing nets), strictly protected Scaup individuals. The authority issuing the relevant permit for this study was the Ministry of the Environment, Warsaw, Poland (2008, permit No: DLOPiK-op/ogiz-4200/III-6/583/08/wo) and the Regional Environmental Conservation Directorate in Szczecin, Poland (2009–2013, permits Nos: RDOŚ-32-WOPN-6631/z/2/10/09/mk; RDOŚ-32-WOPN-6631/z/D/12/10/mk; WOPN.6402.100.2011.MK; WOPN.6401.186.2012.AA). The vertebrate work did not have to be approved by any Institutional Animal Care and Use Committee (IACUC) or equivalent animal ethics committee, because no protected species were sampled specifically for this study.

## Results

### Spatial distribution and biomass of Zebra Mussels

**Fig 4:**
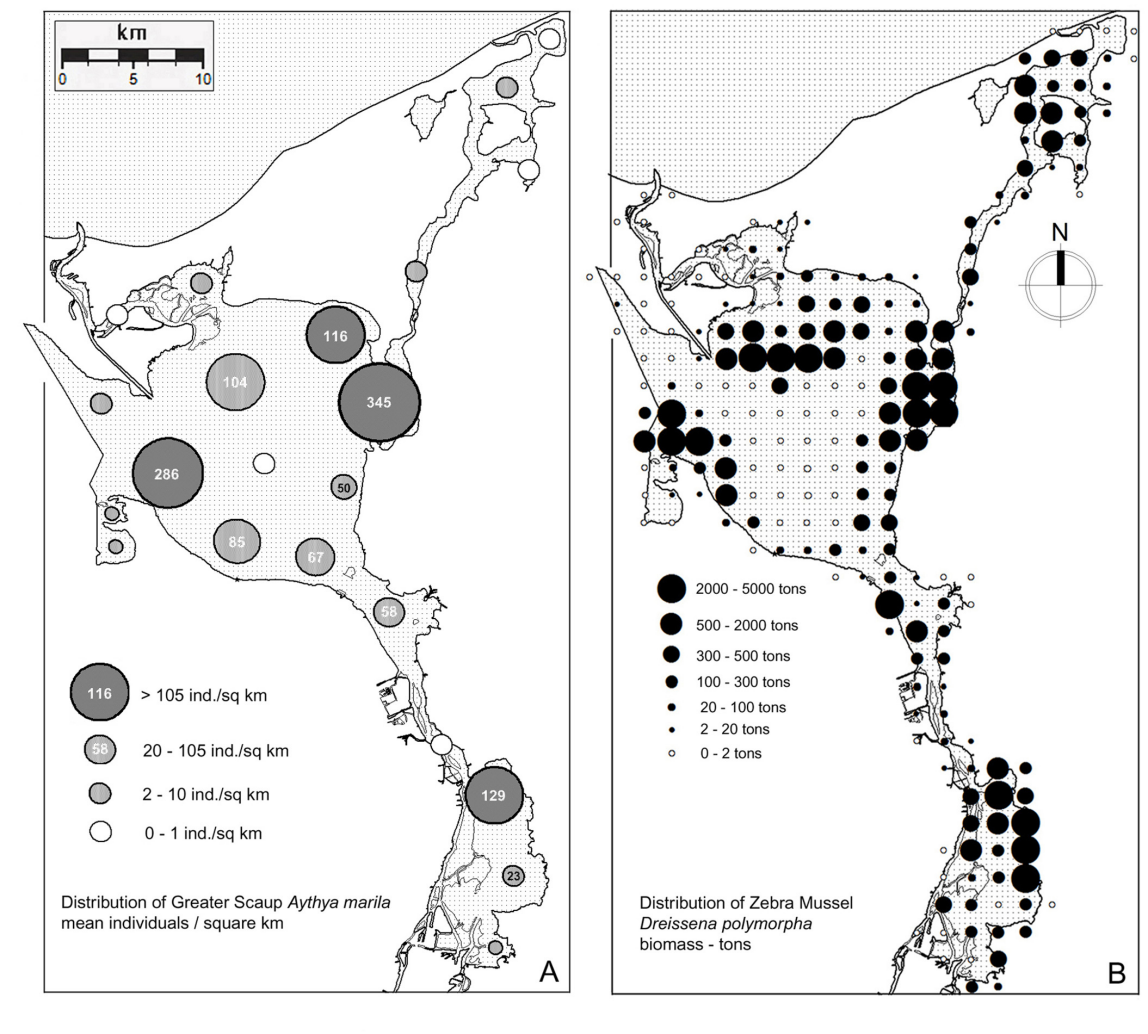
Spatial distribution and abundance of Greater Scaup *Aythya marila*–mean number of individuals per 1 km² based on censuses in the non-breeding period (October to April, 2002–2014), see also Table 2. (A). Spatial distribution and biomass of Zebra Mussel *Dreissena polymorpha* on the 2x2 km modeling grid (B).

The spatial distribution of the Zebra Mussel biomass in the ORE was highly uneven (Fig 4B). The sampling point with the highest biomass was recorded in the central-eastern part of Lake Dąbie (DLC, 36 107 g / m^2^). Other distinctive areas with a high biomass of Zebra Mussels were the northern Lake Dąbie (DLN), Roztoka (RO), Skoszewska Cove (SKC), Miroszewo (MI) and Krzecki Wyskok (KW). The largest area nearly devoid of mussels was the central part of the Szczecin Lagoon—ML.

147 of the 209 ORE 2x2 km squares were occupied by the Zebra Mussel, and its occurrence was predicted in a further 8 squares (not sampled); in total, 155 squares (74%) were considered as being occupied by the mussels. 33 of the squares surveyed were unoccupied, and we predicted the absence of mussels in a further 21 squares (not sampled); therefore, a total of 54 squares were assumed to be unoccupied by the mussels. The highest biomass was calculated for a square in the northern part of Lake Dąbie (DLN)– 5 436 tons (per 4 km^2^), a value very similar to that for a square in central Skoszewska Cove (SKC)– 5 364 tons. Biomasses >5 000 tons were also calculated for single squares on Krzecki Wyskok (KW) and in the northern Skoszewska Cove (SKC). Four subareas were characterized by a particularly high Zebra Mussel biomass—the Skoszewska Cove (SKC), Krzecki Wyskok (KW), Northern Lake Dąbie (DLN) and Miroszewo (MI)–where approximately 72% of the total Zebra Mussel resources in the ORE were found (Fig 4B). The only area where there were no Zebra Mussels was Cicha Cove (CC), most likely because the bottom was unsuitable. Other areas with a low mussel biomass included Lake Wicko (WL), the Świna River (SW), Lake Nowowarpieńskie (NWL) and Lake Wrzosowo (LW) (Fig 4B). The total Zebra Mussel biomass in the ORE was estimated at 94 280 tons.

### Numbers and spatial distribution of Scaup

At the subarea level, the mean number of Scaup recorded during counts without ice cover was 1 330 birds and the highest count was 60 400 individuals (KW subarea, November 21, 2013) (Table 2). In the whole ORE, the three peak counts were recorded in April 2011 (105 700 birds), in October 2013 (97 800) and in November 2013 (80 400). During most censuses, the vast majority of birds were present in nine subareas out of 22, which on average accounted for 90.4% (range 76.5%–98.2%) of the Scaup numbers recorded in the entire ORE during a given census (Fig 4A). In sheltered subareas, we found small numbers of birds or none at all, whereas the largest numbers congregated in areas not sheltered from the wind and exposed to considerable wave action, including Skoszewska Cove, Miroszewo and Krzecki Wyskok. These two groups of subareas had significantly different mean numbers of birds (mean_sheltered_ 4.5 ± 1.53 SE, 95% CI: 1.7–7.7; mean_exposed_ 71.4 ± 21.29 SE, 95% CI: 33.9–116.6; P value from the permutation test: 0.0266).

**Table 2:** Distribution of Scaup in 22 subareas of the Odra River Estuary in 2002–2014 during the non-breeding period (October–April). N—number of counts, %FO—frequency of occurrence during a given number of counts in the subarea, D—mean density (mean number of individuals per km^2^), Mean—mean number of individuals, Min.—minimum number of individuals, Max.—maximum number of individuals (see S4 Table).

Subarea	N	Mean	Min.	Max.	%FO	D
Miroszewo (MI)	35	5 842	0	27 000	85.7	286.4
Krzecki Wyskok (KW)	28	6 017	0	60 400	78.6	103.9
Skoszewska Cove (SKC)	30	5 758	0	37 800	80.0	344.8
Sułomino (SU)	29	3 293	0	42 600	75.9	115.0
Northern Lake Dąbie (DLN)	28	2 460	0	10 000	92.9	128.8
Roztoka (RO)	32	1 694	0	16 000	81.3	58.2
Czarnocin (CZ)	29	1 234	0	1 950	62.1	50.4
Brzózki (BR)	35	1 021	0	6 507	60.0	85.44
Trzebież (TR)	37	739	0	6 150	73.0	66.9
Central Lake Dąbie (DLC)	26	668	0	5 504	65.4	22.6
Refulat (RE)	29	100	0	2 000	27.6	4.3
Main Lagoon (ML)	28	93	0	1 500	39.3	0.6
Southern Lake Dąbie (DLS)	26	45	0	1 000	19.2	5.8
Dziwna (DZ)	31	68	0	1 000	25.8	5.4
Kamień Lagoon (KL)	30	69	0	700	46.7	2.3
Nowowarpieńska Cove (NWC)	33	57	0	700	30.3	1.0
Lake Nowowarpieńskie (NWL)	33	55	0	700	33.3	8.6
Lake Wicko (WL)	29	24	0	500	20.7	2.2
Odra River (OR)	30	11	0	250	33.3	1.2
Lake Wrzosowo (LW)	30	4	0	80	6.7	0.7
Świna River (SW)	29	2	0	40	17.2	0.2
Cicha Cove (CC)	30	0	0	0	0	0

### Relationship between Scaup density and the area of occurrence and biomass of Zebra Mussels

The best-supported random part of the model had the effects of region and season and winter as a fixed factor (Table 3: model 1). In subsequent steps, this model was simplified by moving the insignificant smoother of the area term (edf = 1.00, implying that the smoother is not needed), including it as a continuous predictor (model 14; note that model 15, which did not include Area, was worse). We further reduced the number of levels of the winter factor from nine to two in a stepwise manner (results not shown), which identified two winters with generally lower numbers of birds, both significantly different from each other (model 16). The removal of Area (model 17) led to an increase of AIC, and so the best model was model 16, with an adjusted R-square of 0.265, no overdispersion and no heterogeneity in residual plots and correct other diagnostics (see S1 File).

**Table 3:** Generalized Additive Mixed Models fitted to the count data of Greater Scaup *Aythya marila* in the Odra River Estuary, NW Poland. Models 1–12 have various combinations of factors and random effects to find the best starting structure, while models 13–14 have been simplified in a stepwise procedure while keeping the random part fixed. The best-supported model (with the lowest AIC) is given in bold. The full model includes season (SEA), a factor with three levels (autumn, winter, spring) identifying phenological period within a given winter; winter (WIN), a two-level factor identifying winters with low and high numbers, and subarea (SAR), a factor identifying one of 22 ORE subareas. Smoothers include Area—the area of a given subarea (both in km^2^), Dpa—the area of the bottom covered by Zebra Mussel aggregations (in km^2^) and Dpd—the density of Zebra Mussels in a given subarea (tons/km^2^).

Model	Fixed part		Random part	AIC	Scale	Adj R-sq
	Factors	Smoothers				
*1*	*WIN*	*Area*, *Dpa*, *Dpd*	*SAR*, *SEA*	*1982*.*38*	*2*.*57*	*0*.*168*
2	(none)	Area, Dpa, Dpd	SAR, SEA	2025.36	2.91	0.148
3	WIN, SEA	Area, Dpa, Dpd	SAR	2047.34	3.19	0.161
4	(none)	Area, Dpa, Dpd	SAR	2092.23	3.61	0.151
5	SEA	Area, Dpa, Dpd	WIN, SAR	2066.81	3.17	0.141
6	(none)	Area, Dpa, Dpd	SAR, WIN, SEA	2023.44	2.77	0.150
7	(none)	Area, Dpa, Dpd	WIN	2181.44	4.47	0.135
8	(none)	Area, Dpa, Dpd	WIN, SAR	2059.49	3.13	0.137
9	WIN, SAR	Area, Dpa, Dpd	SEA	2256.79	5.30	0.052
10	(none)	Area, Dpa, Dpd	(none)	2233.98	5.15	0.153
11	WIN, SEA, SAR	Area, Dpa, Dpd	(none)	2181.35	4.45	0.025
12	SEA, SAR	Area, Dpa, Dpd	WIN	2233.98	5.15	0.153
13 (1)	WIN, Area	Dpa, Dpd	SAR, SEA	1980.39	2.57	0.168
**14 (13)**	**WIN**	**Dpa, Dpd**	**SAR, SEA**	**1978.01**	**2.57**	**0.171**

**Fig 5:**
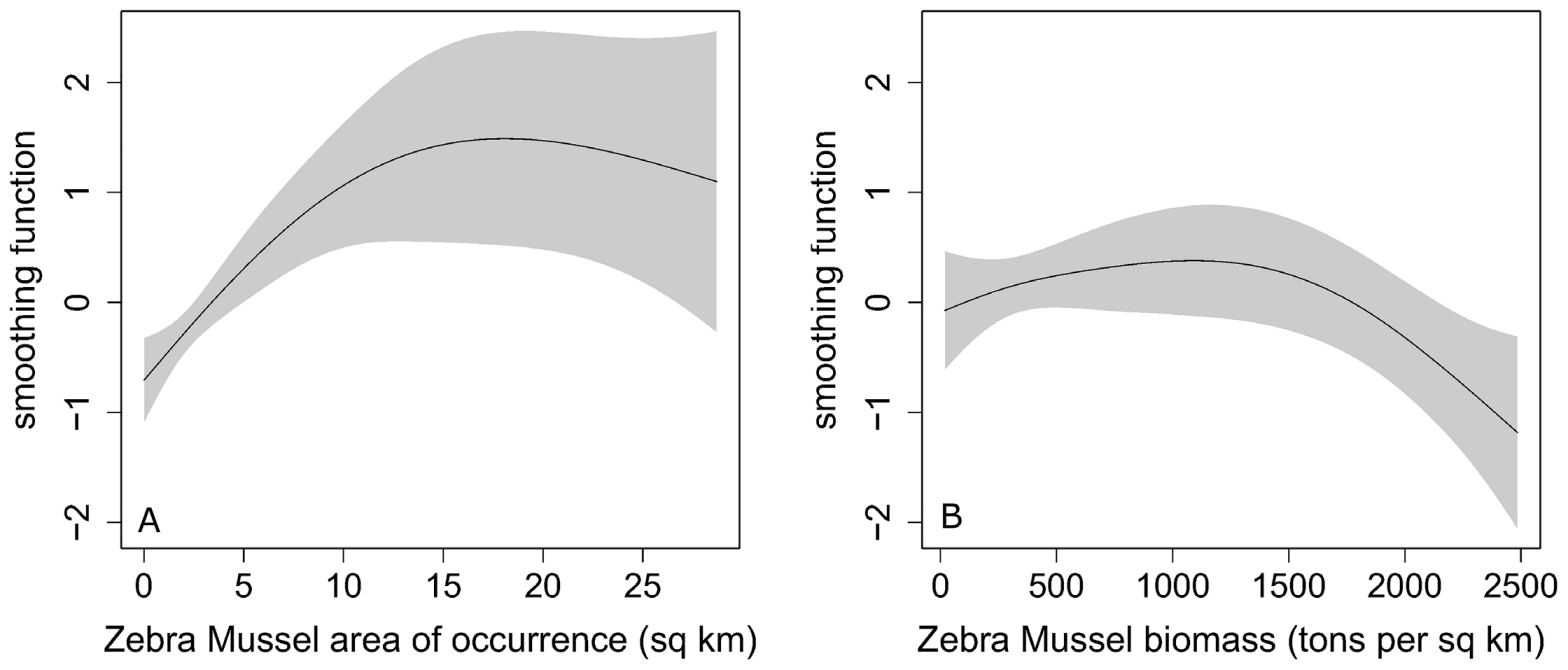
Estimated smoothing functions modeling Greater Scaup *Aythya marila* abundance in relation to the area of occurrence of Zebra Mussel *Dreissena polymorpha* (km^2^, left-hand panel– 5A) and Zebra Mussel density (tons per 1 km^2^, right-hand panel– 5B) from the best-supported model. Shaded areas denote 95% confidence intervals around the smoothing functions.

In all the models considered at any step, both predictors describing Zebra Mussel occurrence (Dpa and Dpd) were highly significant, implying the strong dependence of Scaup density on the extent of occurrence and density of Zebra Mussels (in the final model Dpa: edf = 2.104, F = 14.532, p < 0.0001, Dpd: edf = 2.546, F = 4.844, p = 0.0048). The winter effect (two levels) was also significant (F = 47.83, p < 0.0001). The variance of the random intercept was higher due to subarea (1.037, 95% confidence intervals: 0.678–1.586) than due to season (0.619, confidence intervals: 0.406–0.944), indicating that differences between phenological seasons are smaller relative to the differences between subareas, which is in line with field observations. In all, there was overwhelming evidence that Scaup abundance in the subareas of the ORE depends strongly on the area of occurrence and biomass of Zebra Mussels. The relationship between the Scaup numbers and the area of occurrence of Zebra Mussels increased to reach a maximum at about 15 km^2^ with the mussel aggregations (Fig 5A), while the relationship between duck numbers and mussel biomass showed a clear decline at values higher than 1 500 tons per km^2^ (Fig 5B).

### Food composition

**Fig 6:**
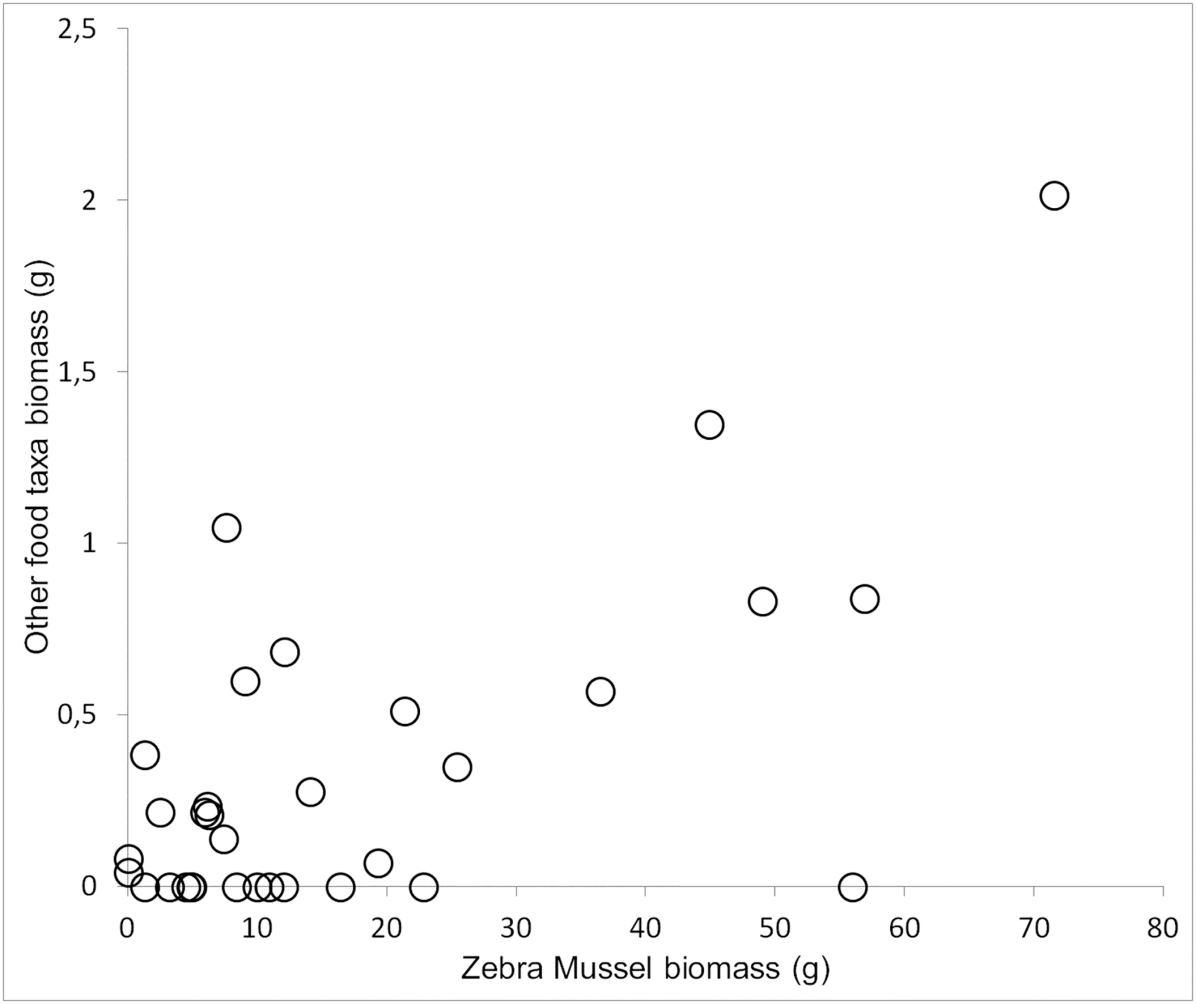
Correlation between Zebra Mussel *Dreissena polymorpha* biomass and biomass of all other food taxa in the digestive tracts of 32 Greater Scaup *Aythya marila* from the Odra River Estuary during the non-breeding seasons in the years 2008–2013. Each point represents a single bird.

The index of feeding success was 0.84 (*n* = 38). Only freshwater taxa occurring in the ORE were found in the digestive tracts of dead Scaup (*n* = 32). We found at least 9 prey taxa. The most important component of the diet found in each food sample (%FO = 100) were bivalves, of which Zebra Mussels appeared the most frequently (%FO = 93.8) and accounted for the largest biomass fraction (%BD = 96.6). We also found other species of bivalves, gastropods, crustaceans, insects and other invertebrates, but even if some were relatively frequent, their biomass was always less than 1% (Table 4). The correlation between Zebra Mussel biomass and the total biomass of all other food taxa was positive and significant (r = 0.66, df = 30, p<0.001) (Fig 6). This indicates that Scaup search specifically for Zebra Mussel and the other prey are eaten only occasionally.

**Table 4:** Composition, abundance, biomass, dominance (%BD) and frequency of occurrence (%FO) of food taxa taken by Greater Scaup *Aythya marila* (n = 32) drowned in fishing nets in the Odra River Estuary (2008–2013) during the non-breeding season.

Taxon		Number of items		Biomass—grams		
	% FO	Mean	Max	Mean	Max	% BD
***Bivalvia* total**	**100.00**	**11.5**	**44**	**17.27**	**71.52**	**96.75**
*Dreissena polymorpha*	93.75	11.1	44	17.23	71.52	96.55
*Sphaeriidae*	21.88	0.5	3	0.04	0.43	0.20
***Gastropoda* total**	**25.00**	**0.8**	**5**	**0.10**	**0.71**	**0.56**
*Theodoxus fluviatilis*	15,63	0.5	5	0.07	0.71	0.38
*Gastropoda* indet	12.50	0.2	3	0.02	0.28	0.11
*Viviparus sp*.	3.13	0.1	2	<0.01	0.39	0.07
***Crustacea* total**	**12.50**	**<0.1**	**3**	**<0.01**	**<0.01**	**<0.01**
*Amphipoda*	12.50	<0.1	3	<0.01	<0.01	<0.01
***Insecta* total**	**40.63**	**2.3**	**23**	**0.16**	**1.61**	**0.88**
*Chironomidae*	40.63	2.3	23	0.16	1.61	0.88
***Annelida* total**	**18.75**	**1.09**	**9**	**0.04**	**0.38**	**0.24**
*Oligochaeta*	18.75	1,06	9	0.04	0.38	0.24
*Hirudinea*	3.13	0,03	1	<0.01	<0.01	<0.01
**unidentified prey**	**62.50**	**2.72**	**8**	**0.28**	**1.87**	**1.56**

### Calculation of yearly food consumption

Taking into account the food composition of Scaup in the ORE– 96.6% of the biomass consists of Zebra Mussels and the daily demand is 1 790 grams of fresh mass (FM)–we calculated the daily Zebra Mussel intake by Scaup at 1 729 grams of fresh mass (excluding the water in the mantle). During a single non-breeding season (180 days) Scaup consume an average (mean count in 2002–2014) of c. 5 400 tons of Zebra Mussels, which makes up 5.6% of the entire Zebra Mussel resources in the ORE. During the mild season (without ice cover), Scaup can consume c. 12 000 tons (as in the 2004/2005 and 2010/2011 seasons), which represent c. 12.5% of the whole Zebra Mussel biomass in the ORE. In contrast, the presence of ice cover, forming quickly during harsh winters, reduces mussel consumption as few birds are then present; for example, in the season of 2007/2008 consumption was calculated at c. 760 tons, i.e., less than 1% of the Zebra Mussel biomass in the ORE (Table 5).

**Table 5:** Yearly consumption of Zebra Mussels by Scaup in the Odra River Estuary between 2002 and 2014.

Season	Seasonal mean number of Scaup (ind.)	Yearly food consumption (tons)	Percentage of the Zebra Mussel resource in the ORE
2002/2003	7 190	2 240	2.4
2003/2004	8 061	2 510	2.7
2004/2005	37 890	11 790	12.5
2005/2006	3 099	964	1.0
2007/2008	2 455	764	0.8
2008/2009	21 013	6 540	6.9
2009/2010	7 903	2 460	2.6
2010/2011	38 156	11 870	12.6
2011/2012	17 370	5 406	5.7
2012/2013	20 187	6 280	6.7
2013/2014	28 194	8 770	9.3
**Mean**	**14 847**	**5 418**	**5.6**

## Discussion

The Odra River Estuary is a key site for the Scaup population wintering in Europe. The Scaup’s primary food here is the Zebra Mussel, a fact that had been unknown, though not unexpected, prior to this study. Studies published to date show that Scaup forages mainly on marine mussels in the Baltic area [5, 6, 20]. The results of the present work document for the first time that the Zebra Mussel is the Scaup’s primary prey item in the ORE and that the ducks go to areas where it is widespread (Fig 5A). At the same time, the decline in Scaup numbers with increasing Zebra Mussel biomass (Fig 5B) may look odd at first glance. The most common model describing the distribution of a predator in space and its intake rates is the 'type II' functional response model [40, 41], which predicts intake rates by resource density and competition between predators. Within this model, intake rates increase with prey density until they stabilize at a certain asymptotic value, where they are mostly limited by prey handling time: at high prey densities, predators find their prey immediately (i.e., the time spent searching is minimized due to high prey density). Recent studies of waders indicate that when the density-dependence of prey is negative, the functional response becomes hump-shaped (type IV), as intake rates decline at the highest prey densities [42]. This fits the results reported here, in the Zebra Mussel—Scaup system in the ORE: intermediate mussel densities might be preferred, but the negative density-dependence remains to be demonstrated. In the Zebra Mussel, the highest biomass occurs where the aggregations are the densest, which may mean that ducks forage less efficiently, i.e., they spend little time searching for prey, but have to make a greater effort to take a bivalve. In areas of the highest biomass of bivalves the mussel clusters are bigger, so ducks have to take them up to the surface to crush; consequently, the predator's energy expenditure per consumed prey item is greater. At intermediate densities mussels occur in smaller clusters, so ducks can swallow them under the water. Another factor is that ducks, after having carried larger clumps of mussels up to the surface, run the risk of having their food stolen by parasitic gulls [43]. Hence, it is safer to swallow at once smaller clumps of mussels under water than to bring bigger ones to the surface. Another explanation put forward is that searching efficiency may decline with predator group size [44]. Our simple analysis comparing preferred depths in Zebra Mussel and Scaup (S2 Fig) shows that, in general, both Scaup and Zebra Mussel follow similar depth ranges. In more detail, however, it appears that the biomass of Zebra Mussels at 1–3 m depth (c. 570 ind./m²) [32] is still high enough to meet the Scaup’s nutritional requirements; the ducks do not have to dive deep, thereby saving energy. Although at 3–4.5 m depth there are more Zebra Mussels (1 800 ind./m²) [32], they are less accessible to the ducks—foraging in bigger depths is more costly. There is little information about the depth diving preferences of Scaup: de Leeuw [8] reports that these ducks select the shallowest areas and do not usually dive deeper than 5 meters. According to Mendel et al. [5] larger flocks do not appear beyond the 10 m isobath, while Johnsgard [45] reports that Scaup are capable of diving to a depth of 7.5 m, but usually go only as far as 1.5 m.

The food composition in the ORE is similar to that in the Dutch lakes IJsselmeer and Markermeer [8]. On the basis of digestive tract contents we have shown that Scaup in the ORE feed almost exclusively on Zebra Mussel and probably do not look for other prey. Whether *Sphaeriidae* represent the second target prey requires further study; the fact that these mussels were found in two individuals that had not ingested Zebra Mussels suggests that this might be so. The remaining taxa identified in stomachs were presumably consumed opportunistically, while ducks were foraging for Zebra Mussels. A high frequency of occurrence (%FO = 40.6) of *Chironomidae* larvae was found, but their proportion in the biomass was negligible (%BM = 0.9). The dominance of chironomid larvae in the diet of Scaup overwintering on freshwater Lough Neagh (Ireland) was reported by Winfield & Winfield [46]. The muddy bottom areas of ORE are colonized by a dense population of chironomid larvae, which are one of the two main muddy-bottom benthos taxa here [32]. Scaup may consume them throughout their stay in the area, by taking larvae inhabiting the bottom. It can therefore be assumed that where these larvae occur in large numbers, they may represent a significant secondary source of food, albeit a less accessible one to diving ducks as they have to be taken from deeper, muddy-bottom areas.

The large concentrations of foraging Scaup, present in the ORE for about six months during mild winters, could locally and periodically deplete the population of its primary food—the Zebra Mussel—to a considerable extent. Such an impact of benthivorous divers on the zoobenthos has been demonstrated in previous studies [8, 18, 47–49]. On the other hand, even moderately depleted mussel populations are still able to recover quickly or found new populations, thanks to the dispersal of the planktonic larvae stage [21], so such depletions—assuming that they do occur—probably do not affect the whole ORE Zebra Mussel population in the long term.

Owing to the low energy value of mussels and the large amount of energy expended in the process of acquiring food, a single Scaup must eat a daily quantity of mussels corresponding to three times its body mass [8]. With a mean Scaup density of 345 ind/km^2^ in Skoszewska Cove (Table 2), the average consumption there should be about 10 tons daily and 1 800 tons during a mild season with no ice cover. The evidence suggesting the serious impact of Scaup on the local Zebra Mussel population in the ORE comes from Skoszewska Cove. It was there that the highest-ever number of wintering Scaup in the whole ORE was recorded– 54 000 birds in January 2005 [11], with a calculated mean number of 38 000 birds for the whole 2004/2005 season (Table 5). In 2005, the Zebra Mussel population in Skoszewska Cove was found to have declined by c. 90% [30]. The winter of 2004/05 was mild, without any ice cover, and Scaup were permanently present in the ORE between autumn and spring. Our careful estimates suggest that during mild seasons, Scaup alone (excluding other diving species present, like Tufted Duck, Pochard, Goldeneye *Bucephala clangula* and Coot *Fulica atra*) could consume c. 38% of the Zebra Mussel population in Skoszewska Cove. Unfortunately, because no systematic monitoring of the Zebra Mussel populations was undertaken at that time, a more detailed analysis is not possible. Diving ducks consume mussels 5–30 mm long [8] and exhibit a tendency to concentrate in large, dense flocks in small areas [12–14]. It therefore seems likely that in some years they may locally overexploit food resources. De Leeuw [8] observed a similar situation in the Netherlands, where in a single season flocks of Scaup and Tufted Duck exploited over 90% of the Zebra Mussel resources in some patches of water body bottoms. An overexploited Zebra Mussel population requires a few years to recover [21, 50, 51], so ducks are then forced to seek other foraging areas. This might partly explain the wide fluctuations of Scaup numbers from year to year and between subareas in the same season recorded in the most frequently occupied subareas, e.g. Skoszewska Cove, Miroszewo or Krzecki Wyskok. After a winter with large numbers of Scaup, there were no birds at all during the next winter, despite the absence of ice cover. The mean annual consumption of Zebra Mussels by Scaup in the ORE is 5.6% (0.8%–12.6%), which points to substantial differences in the exploitation of mussels in different years, depending on the severity of the winter. In cold winters Scaup are less abundant and thus the consumption of mussels is lower. On the Dutch lakes IJsselmeer and Markermeer, De Leeuw [8] estimated the yearly consumption of mussels by Tufted Duck and Scaup to be 10%–20% of the whole Zebra Mussel population. Overexploitation of the marine mussel populations has been suggested for other areas as well [47–49]. Long-tailed Ducks *Clangula hyemalis*, Velvet Scoters *Melanitta fusca* and Common Scoters *M*. *nigra* in 2003/2004 led to considerable changes in the foraging areas in the vicinity of Cape Rozewie (southern Baltic Sea, northern Poland), which forced the birds to seek other feeding areas [49]. In another study, Stempniewicz [52] estimated that Long-tailed Ducks consume 6 350 tons of mussels annually in the western part of the Gulf of Gdańsk (southern Baltic, Poland). In the ORE, the Zebra Mussel population seems to be stable and the area of its occurrence has varied only slightly during the course of a few decades [29–30]. The average seasonal abundance of birds in the study period and, calculated on this basis, the average annual consumption of Zebra Mussel varies significantly: periods of high pressure on mussels are interspersed with periods of low consumption (Table 5), which presumably allow the mussel populations to recover. In the context of climate warming and the consequent shorter duration of ice cover on water bodies, however, the pressure of Scaup on Zebra Mussels may increase, which in turn may lead to a gradual reduction of mussel stocks. This has already been recorded in the Netherlands [53].

Local and seasonal changes in the number of Scaup may also be due to the presence or absence of suitable size classes of mussels in a particular place or in a given year or both. Zebra Mussels in the study area are available in a wide range of size classes (4–32 mm), with the vast majority of mussels being 4–22 mm in size [30]. Scaup eats mussels in all size classes occurring in the study area, but prefer the 10–22 mm size class [8]. Selectivity, however, could not be tested by comparing length distributions in the field and in the diets of the ducks because of strong local and annual differences in the length structure of the mussel populations [8].

The large fluctuations in numbers and the highly variable spatial distribution of Scaup in the ORE must also stem from weather conditions: in winter, numbers decline with increasing ice cover. We have deliberately omitted the results of counts made in such conditions, since they would blur the relationship we were looking for while not contributing any interesting results. A number of studies have reported the obvious relationship between decreasing numbers of water birds with increasing ice cover [54–56].

Although the Zebra Mussel is a non-native species [21], it currently plays an important role in the ecosystem of the Szczecin Lagoon [29–30]. Its filtering abilities improve the physicochemical properties of the water [30, 57– 59]. The mussels in the ORE filter the water from an area of 118 861 km^2^ [58] and a wide array of compounds from the filtered water are accumulated by Zebra Mussels into their tissues and shells. They include both nutrients and heavy metals [57–59]. The importance not only of Scaup and other diving benthivorous birds but also of fish like Roach *Rutilus rutilus* [60] and Round Goby *Neogobius melanostomus* [28] in this ecosystem should be considered in the context of improving the water quality of the ORE and the nearby areas of the southern Baltic. Scaup breed mainly in the boreal parts of Russia, in the taiga and tundra zones [1]. By foraging in the ORE from October to April they accumulate trace elements like other diving ducks [61–68], and when migrating to their breeding areas, the birds act as vectors transporting and dispersing trace elements over considerable distances [61]. This means by which waterfowl reduce contaminant burdens may result in the accumulated elements and compounds being deposited in the shells of their eggs [67]. The importance of the duck-mussel system could be much greater than previously thought: it may include the transfer of trace elements between geographical zones, for distances of up to several thousand kilometers.

For a large part of the Scaup population (38%–70%) during the non-breeding season (between October and April) [31], the ORE is not only a resting and feeding area important for accumulating the energy reserves needed for the northward migration. Birds also display, form pair bonds and copulate, and the females form eggs here and therefore, the Zebra Mussel resources in the ORE play an important role in the whole biogeographical population of Scaup.

The factors responsible for the alarming decline in the Scaup population, by 60% over the last two decades, remain unknown [15]. Studies in the near future should aim primarily at determining whether the trace elements contained in the bodies and shells of the Zebra Mussels consumed in the ORE adversely affect the Scaup’s breeding success. Trace elements found in Common and Spectacled Eiders from Alaska [67] or in Scaup from northern Canada [68] did not reduce their breeding success. If this is also the case in the ORE, the Zebra Mussel’s populations should be conserved, even though it is an alien species. This would also suggest that food supply is not a driving factor contributing to the Scaup’s decline. The situation may be further complicated by the recent appearance in the ORE of a new invasive species, the Quagga Mussel *Dreissena rostriformis bugensis* (first record in 2014) [69], which may become an important component of the Scaup diet.

## Supporting Information

S1 Fig Map of the Odra River Estuary with the 2x2 km square grid overlain. One subarea—Skoszewska Cove (SKC) and the seven squares covering it—is highlighted.
(TIF)

S2 Fig Numbers of Scaup and biomass of Zebra Mussel plotted agains mean depth at the level of subregion. Symbols denote means, whiskers—95% confidence intervals. Blue—Scaup numbers, green—Zebra Mussel biomass.
(XLSX)

S1 File Electronic Appendix. The results of Generalized Additive Mixed Models fitted to the count data of Greater Scaup *Aythya marila* in the Odra River Estuary, NW Poland.
(DOCX)

S1 Table All the raw data on the benthos sampling stations. (1) Number. (2) Sampling year. (3) Sampling depth. (4) Zebra Mussel Biomass (g/m²). (5) and (6) Geographical coordinates.
(PDF)

S2 Table Data set on which the analysis was performed. Data from the 22 counts of the Greater Scaup *Aythya marila* in the Odra River Estuary in the period 2002–2014 and data on the occurrence and biomass of the Zebra Mussel *Dreissena polymorpha*. REG—numeric code for the Odra River Estuary subarea, DATE—census date, WINTER_NUM—numeric code for the winter (Nov-Apr), COUNT_NUM—numeric code for the census, AREA—area (sq km) of the given ORE subarea, SEASON—variable identifying the phenological period in a given winter (1—autumn, 2—winter, 3—spring), AM—raw Scaup numbers, AM_d—density of Scaup per 1 sq km, sqrtAM_d—square root of the AM_d.
(XLSX)

S3 Table All raw data on the number of Greater Scaup *Aythya marila* in 22 subareas in 2002–2014. (PDF)

S4 Table Data set of the number of Greater Scaup *Aythya marila* in 22 subareas in 2002–2014. (PDF)
