# Correction: The Importance of Non-Native Prey, the Zebra Mussel *Dreissena polymorpha*, for the Declining Greater Scaup *Aythya marila*: A Case Study at a Key European Staging and Wintering Site

**DOI:** 10.1371/journal.pone.0152543

**Published:** 2016-03-25

**Authors:** Dominik Marchowski, Grzegorz Neubauer, Łukasz Ławicki, Adam Woźniczka, Dariusz Wysocki, Sebastian Guentzel, Maciej Jarzemski

There are errors in the first paragraph of the Results section, “Relationship between Scaup density and the area of occurrence and biomass of Zebra Mussels.” Please see the corrected paragraph here.

The best-supported random part of the model had the effects of region and season and winter as a fixed factor (Table 3: model 1). In subsequent steps, this model was simplified by moving the insignificant smoother of the area term (edf = 1.00, implying that the smoother is not needed), including it as a continuous predictor (model 13). We further reduced the number of levels of the winter factor from nine to two in a stepwise manner (results not shown), which identified two winters with generally lower numbers of birds, both significantly different from each other. The removal of Area (model 14) produced the best model, with an adjusted R-square of 0.171, no overdispersion and no heterogeneity in residual plots and correct other diagnostics (see S1 File).
