# Supplementary figures and images for: The Importance of Non-Native Prey, the Zebra Mussel Dreissena polymorpha, for the Declining Greater Scaup Aythya marila: A Case Study at a Key European Staging and Wintering Site
Source: PLoS One. 2015 Dec 28;10(12):e0145496. doi: 10.1371/journal.pone.0145496 (PMC4692530; doi:10.1371/journal.pone.0145496)

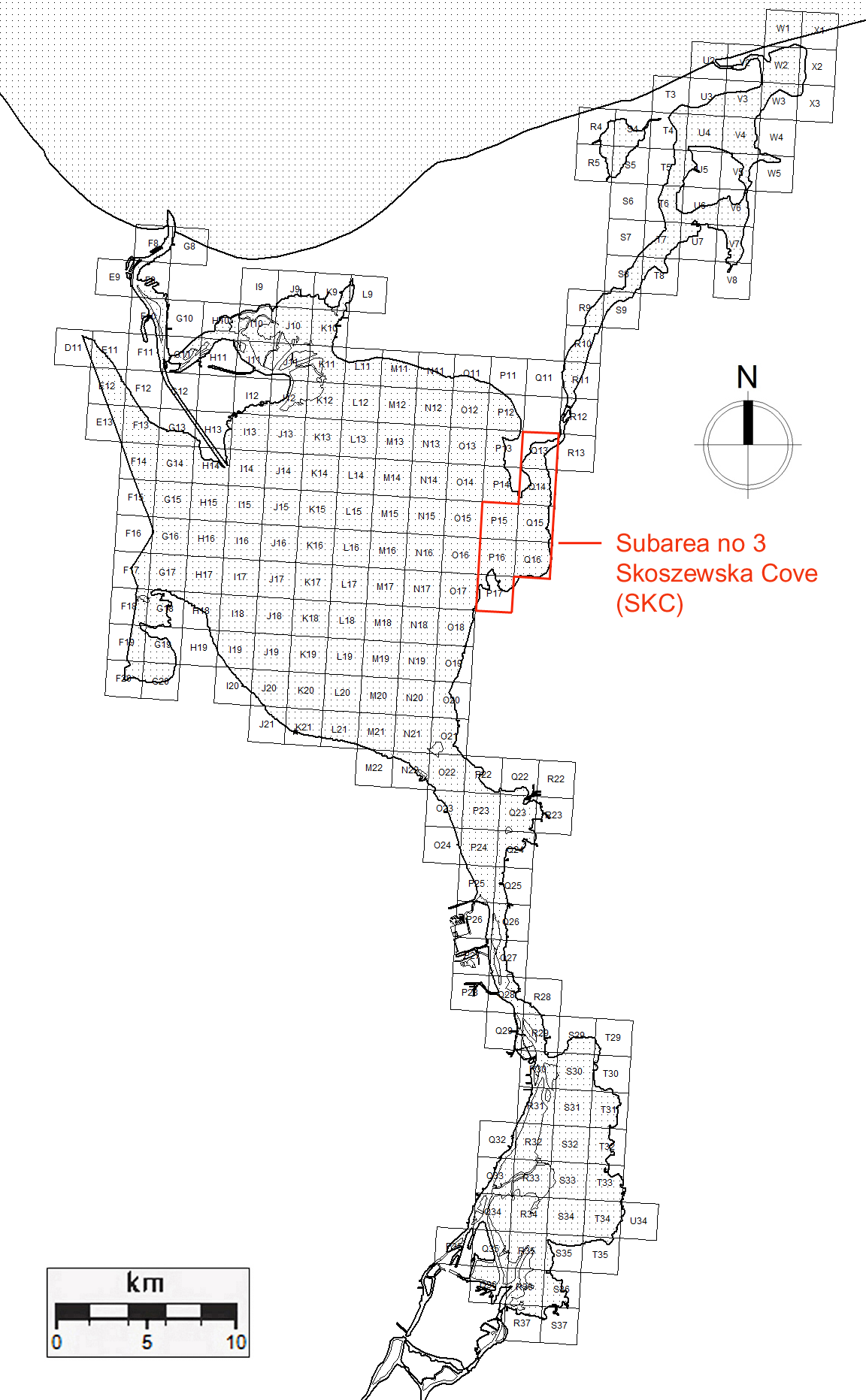

Supplement: S1 Fig — One subarea—Skoszewska Cove (SKC) and the seven squares covering it—is highlighted. (TIF) [file pone.0145496.s001.tif]
