# Supplementary material for: The Importance of Non-Native Prey, the Zebra Mussel Dreissena polymorpha, for the Declining Greater Scaup Aythya marila: A Case Study at a Key European Staging and Wintering Site
Source: PLoS One. 2015 Dec 28;10(12):e0145496. doi: 10.1371/journal.pone.0145496 (PMC4692530; doi:10.1371/journal.pone.0145496)
Supplement: S1 File — The results of Generalized Additive Mixed Models fitted to the count data of Greater Scaup Aythya marila in the Odra River Estuary, NW Poland. (DOCX) [file pone.0145496.s003.docx]

Final model:

gamm2nb.s3 = gamm(sqrtAM_d~WPOOL+AREA+s(DPA)+s(DPD),random=list(REG=~1,SEASON=~1),data=ayt,family=nb(),niterPQL=1000)

Summary gam part:

> summary(gamm2nb.s3$gam)

Family: negative binomial

Link function: log

Formula:

sqrtAM_d ~ WPOOL + AREA + s(DPA) + s(DPD)

Parametric coefficients:

Estimate Std. Error t value Pr(>|t|)

(Intercept) 1.217761 0.197425 6.168 1.47e-09 ***

WPOOLpool2,5 -0.691637 0.130878 -5.285 1.92e-07 ***

AREA -0.006620 0.005633 -1.175 0.241

---

Signif. codes: 0 ‘***’ 0.001 ‘**’ 0.01 ‘*’ 0.05 ‘.’ 0.1 ‘ ’ 1

Approximate significance of smooth terms:

edf Ref.df F p-value

s(DPA) 1.920 1.920 8.966 0.000217 ***

s(DPD) 2.105 2.105 3.125 0.042448 *

---

Signif. codes: 0 ‘***’ 0.001 ‘**’ 0.01 ‘*’ 0.05 ‘.’ 0.1 ‘ ’ 1

R-sq.(adj) = 0.265

Scale est. = 0.7251 n = 48

Summary lme part:

> summary(gamm2nb.s3$lme)

Linear mixed-effects model fit by maximum likelihood

Data: data

AIC BIC logLik

1537.938 1579.759 -758.969

Random effects:

Formula: ~Xr - 1 | g

Structure: pdIdnot

Xr1 Xr2 Xr3 Xr4 Xr5 Xr6 Xr7 Xr8

StdDev: 8.379031 8.379031 8.379031 8.379031 8.379031 8.379031 8.379031 8.379031

Formula: ~Xr.0 - 1 | g.0 %in% g

Structure: pdIdnot

Xr.01 Xr.02 Xr.03 Xr.04 Xr.05 Xr.06 Xr.07 Xr.08

StdDev: 6.010283 6.010283 6.010283 6.010283 6.010283 6.010283 6.010283 6.010283

Formula: ~1 | REG %in% g.0 %in% g

(Intercept)

StdDev: 0.6155125

Formula: ~1 | SEASON %in% REG %in% g.0 %in% g

(Intercept) Residual

StdDev: 0.3298101 0.8515303

Variance function:

Structure: fixed weights

Formula: ~invwt

Fixed effects: list(fixed)

Value Std.Error DF t-value p-value

X(Intercept) 1.2177608 0.1978365 417 6.155388 0.0000

XWPOOLpool2,5 -0.6916366 0.1311510 417 -5.273589 0.0000

XAREA -0.0066197 0.0056452 18 -1.172633 0.2562

Xs(DPA)Fx1 0.5056324 0.3654659 18 1.383528 0.1834

Xs(DPD)Fx1 -0.4067315 0.4938880 18 -0.823530 0.4210

Correlation:

X(Int) XWPOOL XAREA X(DPA)

XWPOOLpool2,5 -0.094

XAREA -0.660 0.002

Xs(DPA)Fx1 0.168 -0.003 -0.261

Xs(DPD)Fx1 0.047 0.002 -0.058 -0.003

Standardized Within-Group Residuals:

Min Q1 Med Q3 Max

-1.1309219 -0.7124418 -0.3528824 0.5052873 4.9612872

Number of Observations: 484

Number of Groups:

g g.0 %in% g REG %in% g.0 %in% g

1 1 22

SEASON %in% REG %in% g.0 %in% g

66

Approximate confidence intervals:

> intervals(gamm2nb.s3$lme, which="var-cov")

Approximate 95% confidence intervals

Random Effects:

Level: g

lower est. upper

sd(Xr - 1) 5.320539 70.20817 884.8633

Level: g.0

lower est. upper

sd(Xr.0 - 1) 0.988794 36.12351 1224.72

Level: REG

lower est. upper

sd((Intercept)) 0.4003035 0.6155125 0.946421

Level: SEASON

lower est. upper

sd((Intercept)) 0.1995165 0.3298101 0.5451914

Within-group standard error:

lower est. upper

0.7959177 0.8515303 0.9110286

Plots of normalized residuals (accounting for the random effects vs predictors):

> E = resid(gamm2nb.s3$lme, type = "normalized")

> plot(ayt$DPA, E)

> plot(ayt$DPD, E)

> plot(ayt$AREA, E)

Significance of smoothing terms:

> anova(gamm2nb.s3$gam)

Family: negative binomial

Link function: log

Formula:

sqrtAM_d ~ WPOOL + AREA + s(DPA) + s(DPD)

Parametric Terms:

df F p-value

WPOOL 1 27.927 1.92e-07

AREA 1 1.381 0.241

Approximate significance of smooth terms:

edf Ref.df F p-value

s(DPA) 1.920 1.920 8.966 0.000217

s(DPD) 2.105 2.105 3.125 0.042448

Plots of smoothing functions:

plot(gamm2nb$gam,select=1,rug=FALSE,xlab="Zebra Mussel area of occurrence (sq km)",ylab="smoothing function",shade=TRUE,shade.col="gray80")
