# Supplementary material for: The Importance of Non-Native Prey, the Zebra Mussel Dreissena polymorpha, for the Declining Greater Scaup Aythya marila: A Case Study at a Key European Staging and Wintering Site
Source: PLoS One. 2015 Dec 28;10(12):e0145496. doi: 10.1371/journal.pone.0145496 (PMC4692530; doi:10.1371/journal.pone.0145496)
Supplement: S1 Table — (1) Number. (2) Sampling year. (3) Sampling depth. (4) Zebra Mussel Biomass (g/m²). (5) and (6) Geographical coordinates. (PDF) [file pone.0145496.s004.pdf]

No (1)	Year (2)	Depth (3)	Biomass (4)	lat (5)	long (6)
1	2003	4.9	0	53.74330000	14.50470000
2	2003	5.3	0	53.73580000	14.47840000
3	2003	5.3	0	53.77280000	14.52340000
4	2003	5.1	0	53.70830000	14.39400000
5	2003	4.5	0	53.70270000	14.38370000
6	2003	3.9	11.5603	53.70100000	14.38170000
7	2003	3.5	516.768	53.69920000	14.37750000
8	2003	1.7	0	53.69780000	14.37630000
9	2003	1.5	0	53.72170000	14.34200000
10	2003	3.9	0	53.72000000	14.34220000
11	2003	1.8	0	53.72000000	14.34220000
12	2003	2.7	89.6301	53.72280000	14.34330000
13	2003	3.2	223.45	53.72470000	14.34550000
14	2003	3.6	69.4285	53.72820000	14.34900000
15	2003	4	4.96103	53.73070000	14.35130000
16	2003	4.5	0	53.73120000	14.35300000
17	2003	5	0	53.73270000	14.35420000
18	2003	1.7	16.5297	53.83770000	14.37500000
19	2003	1.9	12.0967	53.83700000	14.36930000
20	2003	1.7	4.11577	53.83580000	14.36200000
21	2003	1.9	54.2886	53.83380000	14.35570000
22	2003	2.1	0	53.83120000	14.35080000
23	2003	1.5	0	53.82670000	14.34980000
24	2003	1.1	10.3535	53.82470000	14.34270000
25	2003	1	15.2584	53.82470000	14.34270000
26	2003	1.5	0	53.81500000	14.34430000
27	2003	2.3	0	53.81300000	14.34780000
28	2003	2.9	1205.07	53.81200000	14.34920000
29	2003	3.5	3372.8	53.80950000	14.35350000
30	2003	3.9	63.6239	53.80580000	14.36030000
31	2003	2.4	0	53.81250000	14.37320000
32	2003	3	0	53.81930000	14.38600000
33	2003	1.5	7.06059	53.83500000	14.41050000
34	2003	2	6.95896	53.84130000	14.42350000
35	2003	1.9	0.0302691	53.84770000	14.42630000
36	2003	2.5	32.3764	53.85600000	14.44350000
37	2003	3	232.377	53.85580000	14.44600000
38	2003	3.5	10.0024	53.85770000	14.44670000
39	2003	4.5	0	53.80470000	14.35380000
40	2003	5	0	53.80320000	14.35980000
41	2003	2	0	53.81880000	14.46780000
42	2003	2.5	3.139	53.81970000	14.45570000
43	2003	5	870.178	53.82130000	14.49130000
44	2003	4.5	153.345	53.82220000	14.48850000
45	2003	4	40.0044	53.82300000	14.48730000
46	2003	3.5	7.70931	53.82280000	14.48750000
47	2003	3	0	53.82550000	14.48150000
48	2003	2.5	151.838	53.84080000	14.45170000
49	2003	5	0	53.69550000	14.42180000
50	2003	4.5	0	53.68850000	14.41530000
51	2003	4	0	53.68320000	14.41200000
52	2003	3.5	0	53.68220000	14.41330000

53	2003	3	0	53.68120000	14.41450000
54	2003	2.5	87.2505	53.68100000	14.41470000
55	2003	2	0	53.68070000	14.41530000
56	2003	1.3	0	53.68120000	14.41620000
57	2003	1.7	0	53.67350000	14.49070000
58	2003	2.4	111.645	53.67700000	14.49220000
59	2003	3	0	53.67930000	14.49950000
60	2003	3.5	0	53.68080000	14.49280000
61	2003	4	0	53.68020000	14.49350000
62	2003	4.5	0	53.68050000	14.49380000
63	2003	5	0	53.68270000	14.49400000
64	2003	5	0	53.80430000	14.35520000
65	2003	4.5	86.7835	53.80820000	14.35130000
66	2003	1.5	16.943	53.82470000	14.34270000
67	2003	3.5	2037	53.79950000	14.41320000
68	2003	5	0	53.85400000	14.46820000
69	2003	4.8	0	53.85870000	14.47200000
70	2003	4.5	0	53.85900000	14.47280000
71	2003	3.5	10.8022	53.85930000	14.47280000
72	2003	2.5	1.56373	53.85930000	14.47270000
73	2003	2	0.224219	53.85970000	14.47320000
74	2003	2	2.68899	53.86170000	14.44170000
75	2003	4.3	0	53.86170000	14.44120000
76	2003	2	733.977	53.86300000	14.41600000
77	2003	1.5	428.761	53.86300000	14.41600000
78	2003	0.7	80.8324	53.86300000	14.41600000
79	2003	2.5	0	53.81670000	14.36880000
80	2003	3.5	903.896	53.80980000	14.35480000
81	2003	3	25.1009	53.82250000	14.38470000
82	2003	1	210.906	53.84830000	14.41780000
83	2003	1.5	0	53.84830000	14.41780000
84	2003	2	0	53.86320000	14.41870000
85	2003	3	7.53522	53.86350000	14.41830000
86	2003	5	0	53.83520000	14.23850000
87	2003	4.5	0	53.84250000	14.23420000
88	2003	4	117.766	53.84720000	14.23200000
89	2003	3.5	0	53.85150000	14.22900000
90	2003	3	0	53.85570000	14.22620000
91	2003	2.5	0	53.85720000	14.22470000
92	2003	2	0	53.86520000	14.22200000
93	2003	1.5	0	53.86150000	14.21980000
94	2003	5	0	53.81500000	14.32670000
95	2003	4.5	0	53.81630000	14.33050000
96	2003	4	0	53.82570000	14.33280000
97	2003	3.5	0	53.81870000	14.26820000
98	2003	3	0	53.81930000	14.26950000
99	2003	2.5	0	53.81950000	14.26980000
100	2003	1.5	0	53.81970000	14.27000000
101	2003	4.5	422.718	53.69220000	14.52080000
102	2003	4	233.859	53.69320000	14.52330000
103	2003	3.5	991.44	53.69320000	14.52480000
104	2003	3	517.16	53.69330000	14.53220000
105	2003	2.5	30.8703	53.69320000	14.52670000

106	2003	2	19.0328	53.69320000	14.52720000
107	2003	1.5	0	53.69320000	14.52770000
108	2003	5	0	53.72650000	14.51730000
109	2003	4.5	4.05441	53.72600000	14.51930000
110	2003	4	695.037	53.72670000	14.52020000
111	2003	3.5	70.0039	53.72700000	14.52070000
112	2003	3	17.9669	53.72770000	14.52870000
113	2003	2.5	0	53.72830000	14.53450000
114	2003	2	76.9789	53.72880000	14.53520000
115	2003	1.5	0	53.72930000	14.53550000
116	2003	3	0	53.72980000	14.53230000
117	2003	5	0	53.75000000	14.51770000
118	2003	4.5	2221.96	53.74980000	14.52700000
119	2003	4	1411.32	53.75020000	14.53300000
120	2003	3.5	125.547	53.75050000	14.53650000
121	2003	3	22.3808	53.74970000	14.53780000
122	2003	2.5	29.4046	53.74980000	14.53850000
123	2003	2	66.1026	53.83350000	14.55570000
124	2003	1.4	0	53.83370000	14.53930000
125	2003	5	77.3028	53.78070000	14.53700000
126	2003	4.5	1703.1	53.78080000	14.53780000
127	2003	4	5.60842	53.78100000	14.53870000
127	2003	3.5	7.6006	53.78170000	14.53900000
128	2003	3	20.5281	53.78220000	14.54050000
129	2003	2.5	6.63099	53.78230000	14.54830000
130	2003	2	0	53.78270000	14.54980000
131	2003	1.5	0	53.78280000	14.54950000
132	2003	5	809.579	53.82480000	14.52330000
133	2003	4.5	962.379	53.82450000	14.52350000
134	2003	4	129.54	53.82420000	14.52380000
135	2003	3.5	8.45542	53.82480000	14.52450000
136	2003	3	6.89266	53.82520000	14.53180000
137	2003	2.5	0	53.82480000	14.53280000
138	2003	2	7.5427	53.82470000	14.52720000
139	2003	1.5	0	53.82470000	14.52820000
140	2003	5.4	0	53.82520000	14.51980000
141	2003	5.5	169.624	53.76450000	14.31950000
142	2003	5	257.057	53.76250000	14.31830000
143	2003	4.5	1140.57	53.75830000	14.31420000
144	2003	4	3057.04	53.75700000	14.31250000
145	2003	3.5	0	53.82320000	14.31220000
146	2003	2.5	0	53.82220000	14.31250000
147	2003	2	0	53.82220000	14.31180000
148	2003	1.5	0	53.74700000	14.30880000
149	2003	1.9	0	53.75120000	14.30850000
150	2003	5	0	53.79720000	14.32300000
151	2003	4.5	229.964	53.79770000	14.32230000
152	2003	4	0	53.79830000	14.32270000
153	2003	3.3	312.781	53.79830000	14.32220000
154	2003	2	0	53.79900000	14.32150000
155	2003	3.5	1.64859	53.76350000	14.27650000
156	2003	3.8	3614.02	53.76080000	14.28000000
157	2003	2.3	7.05918	53.75830000	14.28330000

158	2003	2	0	53.75470000	14.27350000
159	2003	2.3	0	53.80220000	14.30600000
160	2003	1.6	0	53.80620000	14.29270000
161	2003	4.5	1665.86	53.79800000	14.40720000
162	2003	4	1930.42	53.79870000	14.40700000
163	2003	5.5	0	53.78020000	14.27820000
164	2003	5	0	53.77630000	14.28120000
165	2003	5.5	137.173	53.78870000	14.30950000
166	2003	6	0	53.78720000	14.31000000
167	2003	1.9	0	53.80210200	14.30078900
168	2003	1	12.98	53.82703100	14.27151800
169	2003	0.9	591.33	53.86272900	14.44273400
170	2003	0.4	36.57	53.86296200	14.44498800
171	2003	1	0	53.86199200	14.43964100
172	2003	0.5	22.52	53.86325500	14.44818100
173	2003	0.8	0	53.84869700	14.57335600
174	2003	0.7	0	53.85573730	14.54456800
175	2003	0.7	330.41	53.85657100	14.54551700
176	2003	4	0	53.67483400	14.52308800
177	2003	3.8	0	53.67419700	14.52361000
178	2003	1	990.53	53.69391800	14.54197900
179	2003	0.5	96.31	53.69392200	14.54293900
180	2003	1	0	53.69394000	14.54050300
181	2003	0.5	0	53.69392500	14.54387500
182	2003	1	267.19	53.72281300	14.54689000
183	2003	0.5	197.75	53.72273200	14.54745300
184	2003	1	376.6	53.72278200	14.54618700
185	2003	0.5	0	53.72262300	14.54811000
186	2004	2	92.7339	53.88233333	14.38366667
187	2004	1.6	5.11291	53.88316667	14.37850000
188	2004	3	19.4915	53.88766667	14.36383333
189	2004	1.5	0	53.88700000	14.36283333
190	2004	0.5	20.332	53.88700000	14.36283333
191	2004	5.3	9.46002	53.86816667	14.41600000
192	2004	1.5	12.1271	53.86816667	14.41600000
193	2004	2.2	247.26	53.87600000	14.39316667
194	2004	1.5	0	53.87683333	14.38016667
195	2004	1.5	0	53.87550000	14.37033333
196	2004	2	0	53.89216667	14.39383333
197	2004	1.5	0	53.89133333	14.40966667
198	2004	1.6	0	53.88650000	14.41816667
199	2004	1.8	0	53.87883333	14.42350000
200	2004	1.7	3.70486	53.87383333	14.42350000
201	2004	1.6	3.75858	53.86716667	14.48666667
202	2004	2.5	0	53.87383333	14.34566667
203	2004	1.5	0	53.87383333	14.34566667
204	2004	0.5	0	53.87383333	14.34566667
205	2004	3.5	0	53.87383333	14.34566667
206	2004	5.5	0	53.87383333	14.34566667
207	2004	3	2.76513	53.88433333	14.37266667
208	2004	1	654.9	53.88433333	14.37266667
209	2004	1.9	0	53.89366667	14.43466667
210	2004	1.4	0	53.89716667	14.43616667

211	2004	1	0	53.89850000	14.43050000
212	2004	1.5	0	53.89100000	14.42650000
213	2004	1	0	53.89100000	14.42650000
214	2004	1.5	14.2966	53.89033333	14.43033333
215	2004	5.5	0	53.85800000	14.36016667
216	2004	10	0	53.86883333	14.35050000
217	2004	6	0	53.85816667	14.28683333
218	2004	1.5	0	53.86033333	14.29350000
219	2004	4	0	53.86033333	14.29350000
220	2004	4.5	0	53.85816667	14.29566667
221	2004	2	0	53.85616667	14.29766667
222	2004	6.5	0	53.87266667	14.32283333
223	2004	10	3.24871	53.87216667	14.34500000
224	2004	7.9	0	53.87116667	14.34466667
225	2004	2.8	0	53.86950000	14.34433333
226	2004	2	0	53.86866667	14.34383333
227	2004	1	16.3948	53.86866667	14.34383333
228	2004	2	0.775638	53.86133333	14.39650000
229	2004	5.6	4.72366	53.86083333	14.39716667
230	2004	4	5.19819	53.86000000	14.48100000
231	2004	2	6.14808	53.86000000	14.48100000
232	2004	1.4	0	53.87383333	14.40633333
233	2004	2.2	61.4511	53.87516667	14.40400000
234	2004	2.3	92.0704	53.87600000	14.38966667
235	2004	1.8	0.903181	53.87566667	14.38333333
236	2004	2.1	656.779	53.88000000	14.38300000
237	2004	3.7	0	53.74616667	14.27366667
238	2004	1.7	0	53.73716667	14.27533333
239	2004	1.9	0	53.73650000	14.28166667
240	2004	2.7	0	53.73833333	14.28500000
241	2004	1.7	45.8453	53.74066667	14.28583333
242	2004	1.2	463.285	53.74066667	14.28583333
243	2004	0.6	28.3301	53.74066667	14.28583333
244	2004	1.5	0	53.74066667	14.28583333
245	2004	1.8	0	53.73683333	14.29900000
246	2004	1.5	0	53.73683333	14.29900000
247	2004	1	168.899	53.73683333	14.29900000
248	2004	1.4	0	53.73716667	14.30216667
249	2004	2.1	0	53.73350000	14.29233333
250	2004	2	0	53.72166667	14.27950000
251	2004	1.9	0	53.72216667	14.28116667
252	2004	0.5	17.071	53.72216667	14.28116667
253	2004	0.7	0	53.72216667	14.28116667
254	2004	1.3	0	53.72216667	14.28116667
255	2004	4.1	0	53.82983333	14.61500000
256	2004	2.5	0	53.83433333	14.61850000
257	2004	4.5	119.709	53.84183333	14.61933333
258	2004	2.5	76.022	53.85600000	14.62533333
259	2004	3.2	1190.01	53.85716667	14.62650000
260	2004	2.2	620.238	53.85766667	14.62833333
261	2004	2	841.194	53.85816667	14.62866667
262	2004	1	0	53.85816667	14.62866667
263	2004	4.2	0	53.90116667	14.65633333

264	2004	3	0	53.90183333	14.65350000
265	2004	1.9	223.362	53.90316667	14.65100000
267	2004	1.7	875.524	53.90400000	14.65166667
268	2004	1	28.5363	53.90400000	14.65166667
269	2004	1.2	0	53.90400000	14.65166667
270	2004	0.7	0	53.90400000	14.65166667
271	2004	2.8	9.66992	53.91433333	14.67533333
272	2004	2.2	0	53.93416667	14.70200000
273	2004	2.5	0	53.94983333	14.70883333
274	2004	1.8	1098.66	53.94966667	14.71200000
275	2004	1.5	973.305	53.94966667	14.71200000
276	2004	1	19.2371	53.94966667	14.71200000
277	2004	2.2	0	53.94216667	14.74583333
278	2004	2.1	0	53.94566667	14.73216667
279	2004	1.9	3462.57	53.94850000	14.72383333
280	2004	2.3	0	53.94400000	14.71516667
281	2004	2.4	17.6248	53.95783333	14.69966667
282	2004	2.4	0	53.95900000	14.70266667
283	2004	1.5	27.0155	53.95916667	14.70450000
284	2004	3.2	0	53.98483333	14.75200000
285	2004	3.1	0	54.00133333	14.75183333
286	2004	3.8	0	54.00583333	14.76816667
287	2004	1.5	0	54.00600000	14.76966667
288	2004	5	0.067258	54.00766667	14.77133333
289	2004	1.5	0	54.00766667	14.77133333
290	2004	1	419.21	54.00766667	14.77133333
291	2004	1	51.6554	54.00766667	14.77133333
292	2004	1	1054.36	54.00766667	14.77133333
293	2004	1.5	0	54.01316667	14.77516667
294	2004	2.4	0	54.01200000	14.79450000
295	2004	1.6	0	54.00933333	14.79850000
296	2004	2.6	0	54.01966667	14.79300000
297	2004	2.1	0	54.03083333	14.78916667
298	2004	4.1	0	54.02366667	14.76816667
299	2004	2	0	54.02416667	14.76666667
300	2004	1.5	0	54.02416667	14.76666667
301	2004	3	0	53.99866667	14.72500000
302	2004	1.5	21.8455	53.99866667	14.72500000
303	2004	1.5	0	53.98150000	14.72266667
304	2004	1	51.6554	53.98150000	14.72266667
305	2004	1	1880.6	53.98150000	14.72266667
306	2004	1	0	53.98150000	14.72266667
307	2004	2	3928.35	53.98283333	14.70083333
308	2004	3.2	0	53.88683333	14.64133333
309	2004	2.5	0	53.88766667	14.63816667
310	2004	4.1	399.996	53.57616667	14.58783333
311	2004	3	29.514	53.57133333	14.58650000
312	2004	10	0	53.56800000	14.58966667
313	2004	2	0	53.55416667	14.59783333
314	2004	4	5.15052	53.55900000	14.60216667
315	2004	3	0	53.57200000	14.59416667
316	2004	4.1	386.365	53.60150000	14.59283333
317	2004	2.7	352.409	53.60233333	14.59616667

318	2004	4	74.3316	53.60600000	14.58600000
319	2004	2.7	309.202	53.60650000	14.58200000
320	2004	2.5	17.1808	53.60566667	14.57350000
321	2004	2.1	1120.68	53.61916667	14.57300000
322	2004	3.6	0	53.63250000	14.58083333
323	2004	3.7	0	53.64150000	14.58850000
324	2004	3	660.609	53.64583333	14.60400000
325	2004	2.8	83.9566	53.64750000	14.60900000
326	2004	2	0	53.65000000	14.61000000
327	2004	1.2	52.3933	53.64750000	14.60900000
328	2004	1.5	415.793	53.64750000	14.60900000
329	2004	3	1263.57	53.64250000	14.58850000
330	2004	5.1	0	53.65133333	14.57466667
331	2004	4	0	53.65500000	14.55933333
332	2004	2.5	21.5935	53.66116667	14.56433333
333	2004	2.7	35.4766	53.66316667	14.56766667
334	2004	4	169.71	53.66016667	14.54583333
335	2004	3.8	930.651	53.65850000	14.54383333
336	2004	3.4	178.393	53.65716667	14.54016667
337	2004	2.2	8.46609	53.65600000	14.54233333
338	2004	3	1629.23	53.64633333	14.55233333
339	2004	2.4	528.615	53.64233333	14.55233333
340	2004	3.2	602.058	53.63466667	14.56800000
341	2004	3.8	197.571	53.53366667	14.62983333
342	2004	1.9	1001.72	53.53366667	14.62900000
343	2004	1.5	2.69506	53.53366667	14.62900000
344	2004	2.5	0	53.50433333	14.62700000
345	2004	1.9	22.2297	53.46183333	14.60116667
346	2004	1.5	0	53.45116667	14.60150000
347	2004	1	0	53.45116667	14.60150000
348	2004	0.7	3.65032	53.45116667	14.60150000
349	2004	2.5	0	53.44350000	14.61983333
350	2004	2.2	0	53.43716667	14.63733333
351	2004	4.3	0	53.43566667	14.63916667
352	2004	2.3	0	53.41733333	14.64516667
353	2004	1.6	23.2849	53.40933333	14.64450000
354	2004	2.5	20.5258	53.40133333	14.64266667
355	2004	2.4	20.6634	53.43766667	14.64450000
356	2004	2.1	171.991	53.45533333	14.64366667
357	2004	1.5	274.555	53.45533333	14.64366667
358	2004	1.5	188.368	53.45533333	14.64366667
359	2004	3	0	53.45766667	14.65400000
360	2004	2.7	4009.24	53.46183333	14.69150000
361	2004	2.7	0	53.46216667	14.69983333
362	2004	2	1891.39	53.46216667	14.70550000
363	2004	1.5	174.547	53.46216667	14.70683333
364	2004	2.3	2310.15	53.47033333	14.68616667
365	2004	2.7	38900.6	53.48050000	14.69116667
366	2004	2.4	866.74	53.48066667	14.69350000
367	2004	1.8	0	53.48066667	14.69350000
368	2004	1.5	384.603	53.48066667	14.69350000
369	2004	1	150.17	53.48066667	14.69350000
370	2004	1.5	156.98	53.48066667	14.69350000

371	2004	3.5	40.5742	53.48116667	14.68733333
372	2004	3.1	0	53.49066667	14.65233333
373	2004	2.8	0	53.49300000	14.64350000
374	2004	2.5	0	53.49366667	14.64033333
375	2004	2.7	1442.3	53.50266667	14.65333333
376	2004	2	2833.21	53.51633333	14.65116667
377	2004	3	255.862	53.51766667	14.65633333
378	2004	3.1	932.765	53.51866667	14.66433333
379	2004	2.5	2256.85	53.51933333	14.67183333
380	2004	2.5	71.1954	53.51966667	14.67666667
381	2004	2.3	713.538	53.52550000	14.66900000
382	2004	3.3	279.583	53.52466667	14.65166667
383	2004	8.3	0	53.51466667	14.63766667
384	2004	5.8	1001.6	53.51583333	14.63816667
385	2004	5	0	53.87400000	14.28200000
386	2004	4	0	53.87366667	14.28266667
387	2004	3	0	53.87366667	14.28266667
388	2004	2.5	0	53.87366667	14.28266667
389	2004	1	12.8503	53.87366667	14.28266667
390	2004	3.1	8.40567	53.86816667	14.39183333
391	2004	1.8	1.42741	53.86966667	14.39416667
392	2004	4	0	53.86466667	14.38683333
393	2004	2	2.05345	53.88983333	14.41150000
394	2004	2	18.5007	53.89216667	14.40250000
395	2004	1.8	11.4134	53.89283333	14.38983333
396	2004	2.7	0	53.88150600	14.42235500
397	2004	1.7	30.0786	53.88324300	14.34567900
398	2004	1	19.9987	53.88247500	14.34377700
399	2004	0.7	0	53.88232400	14.34158200
400	2004	3	25.2573	53.88068500	14.34377100
401	2004	1.5	11.2188	53.88048000	14.34526300
402	2004	1	3.52339	53.88115700	14.37215500
403	2004	2.5	0	53.88218700	14.36547000
404	2004	3	0	53.88113200	14.35737400
405	2004	2.7	6.51132	53.83877600	14.37940300
406	2004	2	14.4246	53.83959600	14.38157900
407	2004	0.7	91.1269	53.84049600	14.38348100
408	2004	3	0	53.84139800	14.38592600
409	2004	4.5	0	53.84351100	14.38781300
410	2004	1.5	131.161	53.84537800	14.38915400
411	2004	2.8	8.66478	53.84667400	14.38954500
412	2004	2.5	10.8601	53.85282400	14.39000300
413	2004	1	0	53.85491300	14.38737300
414	2004	0.8	405.384	53.85586800	14.38434900
415	2004	7.5	0	53.86725500	14.28163300
416	2004	3	0	53.86555300	14.28111500
417	2004	1	47.2855	53.86578500	14.27933200
418	2004	9.2	0	53.85933000	14.28190600
419	2004	3.3	0	53.85899100	14.29299400
420	2004	1.5	0	53.86567100	14.42322900
421	2004	1.5	0	53.86736300	14.42170000
422	2004	0.5	0	53.86581600	14.41966900
423	2004	1	163.016	53.86603900	14.41569700

424	2004	0.5	35.3721	53.86667000	14.41226700
425	2004	1.5	48.0677	53.88848000	14.42573800
426	2004	1.5	0	53.88931600	14.43140600
427	2004	0.7	27.4478	53.88937800	14.42743700
428	2004	0.5	23.1191	53.88898100	14.42894900
429	2004	1	0	53.88789300	14.42985300
430	2004	2	0	53.89706300	14.43249700
431	2004	1	20.5046	53.90220200	14.43533750
432	2004	1	128.162	53.90099200	14.43514600
433	2004	1	0	53.89674500	14.43684000
434	2004	1	305.25	53.89978900	14.43639000
435	2004	1.3	0	53.89356600	14.42935100
436	2004	1	0	53.89221500	14.42986100
437	2004	1	58.4919	53.89185900	14.41125700
438	2004	1.5	15.3239	53.88888400	14.41588300
439	2004	1	65.6825	53.87359500	14.38325200
440	2004	1.5	0	53.87167300	14.38557200
441	2004	1	30.3095	53.86864300	14.31740400
442	2004	1	133.135	53.87074700	14.28022200
443	2004	1	147.3	53.72612300	14.28285600
444	2004	2	0	53.72488300	14.27765900
445	2004	5	322.713	53.83937700	14.61916400
446	2004	1.5	0	53.98012900	14.76696900
447	2004	1.5	0	53.97880100	14.76980500
448	2004	1.2	1651.48	53.97713600	14.77128500
449	2004	1	318.326	53.97734600	14.77671500
450	2004	2	0	53.97602400	14.78317100
451	2004	1.2	0	53.65769700	14.52840300
452	2004	0.7	65.8423	53.65598200	14.53336200
453	2004	0.5	0	53.65947100	14.52164500
454	2004	1.5	386.657	53.55635400	14.59644900
455	2004	1	111.94	53.55438900	14.59730700
456	2004	1	256.772	53.55330000	14.59983800
457	2004	0.5	24.3458	53.55210800	14.60343400
458	2013	6	0	53.78990000	14.44650000
459	2013	5.9	0	53.82289200	14.50630800
460	2013	6.2	0	53.78654000	14.41110900
461	2013	3.3	0	53.59470700	14.59063000
462	2013	3	0	53.57804500	14.59362000
463	2013	4.2	0	53.54979400	14.61596300
464	2013	3.2	0	53.54424200	14.62717600
465	2013	3.5	0	53.51262300	14.65951500
466	2013	2.9	0	53.44689200	14.62533700
467	2013	3.5	0	53.45341800	14.65089100
468	2013	2.1	0	53.46477400	14.65566200
469	2013	3	0	53.47967700	14.65038800
470	2013	3	0	53.41185500	14.65077400
471	2013	3.3	0	53.42496200	14.64225700
472	2013	2	0	53.43107800	14.64131900
473	2013	1.8	0	53.43398500	14.61387000
474	2014	1	488	53.73723200	14.30063000
475	2014	0.5	500	53.74138700	14.29494000
476	2014	1.2	0	53.74481700	14.30861100

477	2014	0.2	150	53.74259500	14.30729600
478	2014	1.5	0	53.68907900	14.39566500
479	2014	2	0	53.69023300	14.39696000
480	2014	2.8	0	53.69180100	14.39914700
481	2014	3.2	0	53.69304000	14.40120000
482	2014	4.1	0	53.69444100	14.40290600
483	2014	4.4	0	53.69563500	14.40406200
484	2014	4.7	0	53.69650600	14.40612100
485	2014	2	1798	53.75345000	14.31051400
486	2014	6	0	53.76769500	14.32349700
487	2014	0.5	79	53.89056600	14.42708800
488	2014	0.7	210	53.88954400	14.42603700
489	2014	0.2	79	53.89269400	14.42664900
490	2014	1.7	120	53.89033200	14.42881300
491	2014	0.5	79	53.89647800	14.42807300
492	2014	2.4	0	53.89504500	14.43227400
493	2014	1.5	145	53.85329300	14.52341400
494	2014	1.4	159	53.85271000	14.49901600
495	2014	2.6	87	53.84434700	14.53874400
496	2014	2.6	201	53.83935000	14.54895100
497	2014	2.1	151	53.85268200	14.54045400
498	2014	0.5	95	53.85497200	14.55713600
499	2014	0.7	95	53.85267000	14.56322100
500	2014	1.2	153	53.85904000	14.53186700
501	2014	2.1	95	53.84273700	14.57462300
502	2014	1.5	154	53.83422800	14.57831100
503	2014	0.8	150	53.86086900	14.50498000
504	2014	1.4	0	53.83656100	14.52867900
505	2014	1.3	412	53.67049800	14.52977800
506	2014	4.5	403	53.67042000	14.53779200
507	2014	5.5	400	53.66973900	14.53706600
508	2014	5.2	430	53.66812200	14.54450300
509	2014	3.1	152	53.67772900	14.52474500
510	2014	0.8	0	53.97425900	14.75486500
511	2014	0.5	0	53.97792100	14.74597300
512	2014	0.9	570	53.98035700	14.70981500
513	2014	1.1	572	53.97648500	14.71049900
514	2014	1.5	329	53.92765700	14.68253400
515	2014	1.2	331	53.91724200	14.66915300
516	2014	1.8	0	53.92383400	14.67724200
517	2014	1.5	0	53.90603500	14.65911200
518	2014	0.8	0	53.89302100	14.63541100
519	2014	1	0	53.89007700	14.63467200
520	2014	0.9	301	53.87366200	14.62220400
521	2014	0.9	359	53.87898000	14.62945800
522	2014	1.2	298	53.88850600	14.63402200
523	2014	0.9	329	53.86571400	14.62061600
524	2014	1.8	362	53.86346600	14.62295900
525	2014	0.5	331	53.85600600	14.62070400
526	2014	0.5	15	53.72669000	14.30118200
527	2014	0.5	25	53.72669000	14.30118200
528	2014	6	0	53.78080500	14.31605000
529	2014	6.3	0	53.77542500	14.31810800

530	2014	5.6	0	53.78269500	14.28748800
531	2014	5.7	0	53.77501200	14.29484300
532	2014	4.9	0	53.79201000	14.28357500
533	2014	1.6	0	53.79647000	14.29523400
534	2014	4.1	0	53.80087500	14.26962100
535	2014	1.5	320	53.90358300	14.65119000
536	2014	1.5	340	53.89553200	14.63705900
537	2014	3.6	1930	53.80003100	14.56395400
538	2014	3.5	1720	53.78910000	14.56374900
539	2014	3	1800	53.77575400	14.57295400
540	2014	2.7	1630	53.76361300	14.57689400
541	2014	2.3	1940	53.75853600	14.58012500
542	2014	1.9	2200	53.76528800	14.59802800
543	2014	1	1790	53.76777400	14.60913700
544	2014	1.8	2100	53.77754000	14.60665800
545	2014	2.5	890	53.78245100	14.60263700
546	2014	2.1	2300	53.78932100	14.60002600
547	2014	2.1	1790	53.79498100	14.60188200
548	2014	2.6	2400	53.80279300	14.59995800
549	2014	2.4	450	53.79152900	14.58441100
550	2014	3.5	1680	53.78894400	14.57266100
551	2014	3.5	1900	53.77966400	14.58643700
552	2014	3.4	1400	53.77092000	14.59224900
553	2014	3.5	2100	53.77092000	14.59224900
554	2014	2.8	960	53.81947500	14.60085400
555	2014	2.6	990	53.82618400	14.60684200
556	2014	2.1	2000	53.80933900	14.55860400
557	2014	1.7	0	53.80295300	14.56424200
558	2014	1.8	0	53.78284700	14.55442700
559	2014	1.3	0	53.76768200	14.55554200
560	2014	1.3	0	53.76060600	14.55323200
561	2014	1.3	0	53.80186400	14.54992800
562	2014	6	0	53.78727300	14.50555330
563	2014	5.7	0	53.78209000	14.48316100
564	2014	5.3	0	53.77184000	14.46626800
565	2014	5.4	0	53.75255800	14.47382600
566	2014	6.1	0	53.76727700	14.36167900
567	2014	10.6	0	53.77009300	14.39599100
568	2014	5.6	0	53.76725300	14.43165600
569	2014	5.1	0	53.73568100	14.45051700
570	2014	5.3	0	53.71170000	14.48196000
571	2014	4.9	0	53.69984600	14.44399700
572	2014	5.8	0	53.71856000	14.41642800
573	2014	5.7	0	53.73499000	14.38568800
574	2014	5.7	0	53.75077900	14.37782700
575	2014	5.8	0	53.78679900	14.34738100
576	2014	5.2	0	53.79808400	14.34338600
577	2014	6	0	53.77131300	14.34125900
578	2014	6.1	0	53.75106200	14.36001700
579	2014	5.4	0	53.73211240	14.41560400
580	2014	5.9	0	53.75134500	14.41533700
581	2014	5.8	0	53.78737400	14.38492300
582	2014	5.4	0	53.69510400	14.47898800

583	2014	5.1	0	53.71871600	14.44946700
584	2014	5.1	0	53.71946900	14.37257000
585	2014	6.1	0	53.80644900	14.51772400
586	2014	6.1	0	53.80105200	14.48849900
587	2014	5.2	0	53.83987200	14.48866300
588	2014	5.3	0	53.70688400	14.50171100
589	2014	2.3	8700	53.79093000	14.59285000
590	2014	3.4	399	53.77403700	14.59412700
591	2014	3.2	502	53.78166300	14.56541700
592	2014	1.9	0	53.73152800	14.28801200
593	2014	1.7	0	53.70381100	14.28546300
594	2014	2.8	2430	53.80749200	14.39433900
595	2014	4.8	2670	53.81258500	14.47546300
596	2014	4.8	2260	53.80411600	14.45678600
597	2014	5	2500	53.79786700	14.42996900
598	2014	4.8	0	53.79656200	14.39433800
599	2014	5.9	0	53.79548500	14.40517500
600	2014	6.1	0	53.79502700	14.42714300
601	2014	5.7	0	53.80205100	14.45856300
602	2014	6	0	53.80967400	14.47772800
603	2014	5.5	0	53.81680600	14.49292500
604	2014	5.7	0	53.82717200	14.49327700
605	2014	4.3	0	53.80271700	14.38188300
606	2014	3.4	569	53.80292700	14.44406700
607	2014	2.8	611	53.81009200	14.46562300
608	2014	4.8	608	53.81818500	14.48478700
609	2014	4.7	530	53.82993900	14.47938700
610	2014	2.9	598	53.80790200	14.40101900
611	2014	2.6	603	53.81834100	14.39704900
612	2014	2.5	612	53.81110500	14.42007800
613	2014	2	589	53.81115200	14.42978900
614	2014	2.3	21	53.81481500	14.46827000
615	2014	2.6	23	53.81692100	14.47588600
616	2014	2.2	28	53.82217300	14.47024700
617	2014	1.9	0	53.82217700	14.43011700
618	2014	1.8	0	53.82493900	14.41654300
619	2014	2.4	0	53.83138900	14.36914700
620	2014	2.4	0	53.82815800	14.38209600
621	2014	1.6	0	53.83734800	14.39199700
622	2014	2.1	0	53.84273700	14.43414000
623	2014	4.7	0	53.85172100	14.48326000
624	2014	5.8	0	53.86154700	14.42687300
625	2014	2.1	0	53.87513200	14.41106100
626	2014	1.4	0	53.87654800	14.39254500
627	2014	1	0	53.86187200	14.47755600
628	2014	2.5	0	54.01970600	14.79269700
629	2014	3.4	0	54.00115600	14.74928900
630	2014	3.4	0	53.98753000	14.72251200
631	2014	2.2	0	53.94574800	14.74578800
632	2014	2.3	0	53.93558700	14.75287600
633	2014	0.8	0	53.92805900	14.75739000
634	2014	2.8	0	53.93131900	14.69925500
635	2014	2.2	0	53.94151200	14.70620900

636	2014	3.5	0	53.98647000	14.75513300
637	2014	3.3	2550	54.00613000	14.73966200
638	2014	1	2421	53.99844800	14.71782000
639	2014	3	2485	53.99793600	14.70053500
640	2014	2.8	570	53.98408900	14.70191300
641	2014	2.1	572	53.97259500	14.70231400
642	2014	2	550	53.96891500	14.69994400
643	2014	3.1	592	53.95542100	14.69173300
644	2014	0.4	571	53.97036400	14.71479500
645	2014	3	0	53.97599900	14.70732500
646	2014	2.5	0	53.96780000	14.70626400
647	2014	3.1	0	53.99653600	14.70622100
648	2014	3.2	0	53.99695700	14.72476800
649	2014	6.1	0	53.80504600	14.52856100
650	2014	1.2	0	53.81165200	14.54076700
651	2014	1.7	0	53.79023600	14.55122700
652	2014	6.3	0	53.79098500	14.53131800
653	2014	6	0	53.78056700	14.53178900
654	2014	5.2	0	53.83658400	14.50975600
655	2014	1.6	0	53.84471200	14.52275100
656	2014	1.9	0	53.82749200	14.43197300
657	2014	5.3	0	53.76118100	14.50681200
658	2014	5.4	0	53.75978400	14.52425900
659	2014	1.1	0	53.74839700	14.54370900
660	2014	5.1	0	53.73712200	14.51629900
661	2014	5.2	0	53.72217800	14.51537800
662	2014	1.3	0	53.72931000	14.54164600
663	2014	5.2	0	53.70398400	14.51229100
664	2014	5.8	0	53.68916900	14.51481200
665	2014	10.7	0	53.67812500	14.51849800
666	2014	5.2	0	53.72598200	14.51245800
667	2014	4.6	0	53.69125800	14.41756100
668	2014	5	0	53.68563200	14.49509500
669	2014	4.7	0	53.69817300	14.40832900
670	2014	5.3	0	53.73356400	14.35951500
671	2014	6	0	53.76976900	14.32719900
672	2014	3.9	0	53.64886100	14.56834700
673	2014	3.5	0	53.64301200	14.57648800
674	2014	2.5	0	53.65120100	14.59131400
675	2014	3.9	0	53.66076500	14.55266200
676	2014	3	0	53.62852000	14.57769300
677	2014	3.2	0	53.63428500	14.58743700
678	2014	1.3	0	53.63546200	14.60926400
