# Supplementary material for: The Importance of Non-Native Prey, the Zebra Mussel Dreissena polymorpha, for the Declining Greater Scaup Aythya marila: A Case Study at a Key European Staging and Wintering Site
Source: PLoS One. 2015 Dec 28;10(12):e0145496. doi: 10.1371/journal.pone.0145496 (PMC4692530; doi:10.1371/journal.pone.0145496)
Supplement: S3 Table — (PDF) [file pone.0145496.s006.pdf]

No	shortc	Subarea	km²	JAN02	NOV02	JAN03	MA03	NOV03	JAN04	MA04	NOV04	JAN05	MA05	NOV05
1	WL	Lake Wicko	11.06	0	15	0	0	0	0	0	0	0	?	?
2	SW	Świna River	12.75	0	40	0	0	0	0	4	0	0	?	?
3	SCK	Skoszewska Cove	16.7	40	4500	0	2500	0	0	5300	5000	50	25000	?
4	SU	Sułomino	28.4	0	0	0	0	0	0	4200	2000	800	4000	?
5	KW	Krzecki Wyskok	57.93	0	1502	0	0	0	0	3000	?	0	2000	?
6	ML	Main Lagoon	154.7	0	0	0	0	0	0	0	?	0	?	?
7	RE	Refulat	23.45	0	0	0	0	0	0	100	?	0	?	?
8	NWC	Nowowarp. Cove	3.32	0	0	0	0	30	0	6	?	150	?	?
9	NWL	Lake Nowowarp.	6.42	0	0	0	0	0	0	0	?	0	?	?
10	MI	Miroszewo	20.4	0	800	0	342	3000	0	44	?	27000	?	?
11	BR	Brzózki	11.95	0	2	0	0	200	0	0	?	3000	?	?
12	TR	Trzebież	11.05	0	0	0	0	130	2	0	?	6150	?	5785
13	CZ	Czarnocin	24.5	0	0	0	500	0	0	2	?	3000	?	?
14	RO	Roztoka	29.1	0	6400	0	1010	2200	0	11	12830	16000	1560	250
15	OR	Odra River	9.2	3	1	57	0	1	0	0	0	0	0	?
16	DLN	Lake Dąbie N	19.1	0	3200	0	600	4000	0	1548	3000	800	3	250
17	DLC	Lake Dąbie C	29.5	0	0	0	100	105	0	0	200	0	?	?
18	CLS	Lake Dąbie S	7.8	0	0	0	0	0	0	300	0	0	?	?
19	DZ	Dziwna	12.6	0	0	0	0	0	0	0	0	1000	?	?
20	KL	Kamień Lagoon	25.3	1	1	1	0	0	0	0	120	0	?	?
21	WL	Lake Wrzosowo	6.05	0	0	0	0	0	0	0	0	0	?	?
22	CC	Cicha Cove	1.3	0	0	0	0	0	0	0	0	0	?	?
		TOTAL	522.88	44	16461	58	5052	9666	2	14515	?	57950	?	6285
		ICE COVER<80%		YES	NO	YES	NO	NO	YES	NO	NO	NO	NO	NO
		AT LEAST		44	16461	58	5052	9666	2	14515	23150	57950	32563	6285

JAN06	APR06	NOV07	JAN08	MA08	N0V08	JAN09	MA09	NOV09	JAN10	APR10	NOV10	JAN11	MA11	APR11
0	0	?	?	?	0	0	0	10	0	0	0	0	0	0
0	0	?	?	?	0	0	0	0	0	0	2	0	0	0
0	1500	?	?	?	80	0	23000	500	0	5000	50	0	6800	37800
0	1500	0	?	?	200	0	5000	400	0	0	10	400	451	42600
0	0	?	?	?	2000	0	7500	2600	0	2000	0	4600	3100	4533
0	0	0	?	?	0	0	0	0	0	10	0	0	100	1500
0	0	0	?	0	0	0		0	0	0	0	0	310	2
0	0	0	?	0	0	0	0	0	0	0	0	0	120	0
0	0	0	?	0	0	0	0	0	0	0	0	0	40	0
0	0	?	?	0	3200	0	2700	9000	0	790	21	0	13907	2500
0	0	?	?	0	0	0	300	0	0	200	0	0	3400	400
10	0	30	?	0	130	0	0	1000	0	800	0	0	450	700
0	0	?	1170	0	700	0	3300	500	0	0	400	0	181	5277
0	0	?	?	4760	30	0	0	0	0	0	500	1	199	50
0	0	?	133	0	0	0	15	0	0	0	0	0	0	0
0	0	0	?	?	900	0	7000	400	0	500	1000	420	10000	7000
0	0	0	?	?	80	0	5504	0	0	0	800	80	750	3000
0	0	0	?	?	0	0	100	0	0	0	330	0	0	350
0	0	0	0	0	400	0	300	0	0	0	0	0	0	0
0	2	0	0	0	250	0	220	0	0	0	0	0	0	21
0	0	0	0	0	50	0	80	0	0	0	0	0	0	0
0	0	0	0	0	0	0	0	0	0	0	0	0	0	0
10	3002	?	1303	?	8020	0	55019	14410	0	9300	2063	5021	39808	105733
YES	NO	NO	NO	NO	NO	YES	NO	NO	YES	NO	NO	NO	NO	NO
10	3002	30	2606	4730	8020	0	55019	14410	0	9300	2063	5021	39808	105733

OCT11	NOV11	DEC11	JAN12	FE12	MA12	NOV12	JAN13	MA13	APR13	OCT13-	OCT13-2	OCT13-3	NOV13-1
0	0	0	2	0	150	500	0	0	0	?	?	0	?
0	0	17	3	0	0	0	0	0	0	?	?	0	?
0	0	205	4500	0	6790	6	0	0	5454	?	?	5600	?
3000	330	5000	600	0	2505	3600	0	0	1500	?	?	2800	?
2852	2760	1104	3000	0	11000	15000	0	0	3500	?	?	30000	?
0	30	92	200	0	92	200	0	0	30	?	?	300	?
0	0	1	7	0	160	330	0	0	0	?	?	0	?
0	100	0	0	0	200	500	0	0	0	?	?	0	?
0	700	0	0	0	50	200	0	0	0	?	?	0	?
3400	19070	3020	0	0	5520	19500	1700	0	15000	4600	700	4280	12020
0	3100	2000	4	0	140	6507	0	0	76	4030	140	5900	44
700	397	3000	0	0	800	60	58	0	2000	1200	472	20	200
0	150	22	3400	0	211	0	0	0	1300	?	?	0	?
320	918	60	69	0	199	855	0	0	250	?	?	0	?
?	10	92	50	0	6	10	0	0	4	?	?	?	?
?	450	?	4500	0	3000	400	608	0	1100	?	?	48900	?
?	150	?	1500	0	1000	75	0	0	62	?	?	?	?
?	0	?	0	0	82	0	0	0	0	?	?	?	?
?	0	0	0	0	300	19	70	0	0	0	?	?	?
?	0	19	200	0	700	0	102	0	172	0	?	?	?
?	0	0	0	0	0	0	0	0	0	0	?	?	?
?	0	0	0	0	0	0	0	0	0	0	?	?	?
10272	28355	14632	18035	0	32905	47762	2538	0	30448	?	?	?	?
NO	NO	NO	NO	YES	NO	NO	NO	YES	NO	NO	NO	NO	NO
10272	28355	14632	18035	0	32905	47762	2538	0	30448	9830	1312	97800	12264

NOV13-2	NOV13-3	NOV13-4	DEC13-1	DEC13-2	JAN14-1	JAN14-2	MA14	APR14
0	?	?	?	0	0	0	20	0
0	?	?	?	0	0	0	0	0
1000	?	?	?	2880	21000	0	11220	0
6600	?	?	?	2000	0	0	6000	6000
60400	?	?	?	20	0	0	3000	5000
50	?	?	?	0	0	0	0	0
0	?	?	?	0	0	0	2000	0
30	0	0	0	0	700	0	35	0
8	5	12	110	20	600	0	60	0
4723	9332	13200	17165	5090	2630	3500	200	0
410	0	71	3200	2600	0	0	0	0
700	1000	220	550	250	130	4000	0	400
50	?	?	?	600	0	0	11950	4000
212	?	?	?	0	3620	700	303	1600
0	?	?	?	0	0	250	0	0
5000	?	?	?	?	6000	200	4000	3200
1105	?	?	?	?	0	250	346	2500
0	?	?	?	?	0	1000	0	0
5	?	?	?	0	0	?	24	0
2	?	?	?	?	0	?	7	250
0	?	?	?	?	0	?	0	0
0	?	?	?	?	0	?	0	0
80295	?	?	?	?	34680	?	39165	22950
NO	NO	NO	NO	NO	NO	YES	NO	NO
80295	10337	13505	21025	13460	34680	9900	39165	22950
